# Gemini surfactant-stabilized cubosomes for enhanced topical delivery of 5-fluorouracil in cutaneous squamous cell carcinoma

**DOI:** 10.1016/j.ijpx.2026.100504

**Published:** 2026-02-06

**Authors:** Ruchira Raychaudhuri, Ajinkya Nitin Nikam, Naitik Jain, Abhisheik Eedara, Neha Kandpal, Rajdeep Ray, Krishnadas Nandakumar, Sai Balaji Andugulapati, Srinivas Mutalik

**Affiliations:** a Department of Pharmaceutics, Manipal College of Pharmaceutical Sciences, Manipal Academy of Higher Education, Manipal 576104, Karnataka, India; b Department of Applied Biology, CSIR-Indian Institute of Chemical Technology (CSIR-IICT), Hyderabad 500007, Telangana, India; c Department of Pharmaceutical Chemistry, Manipal College of Pharmaceutical Sciences, Manipal Academy of Higher Education, Manipal 576104, Karnataka, India; d Department of Pharmacology, Manipal College of Pharmaceutical Sciences, Manipal Academy of Higher Education, Manipal 576104, Karnataka, India

**Keywords:** Non-melanoma skin cancer, Topical delivery, 5-Fluorouracil, Cubosome, Gemini surfactants

## Abstract

Cutaneous squamous cell carcinoma (cSCC) is a prevalent non-melanoma skin cancer. Topical chemotherapy offers a non-invasive alternative to surgical treatments, yet the therapeutic efficacy of conventional agents like 5-fluorouracil is hindered by poor skin permeability and systemic side effects. In this study, we developed a gemini-surfactant-stabilized cubosomal gel (OF12-GEL) for enhanced topical delivery of 5-fluorouracil. The optimized cubosomes (OF12) exhibited a particle size of 134.4 nm, PDI 0.23, zeta potential +76.1 mV, and 51.3% entrapment efficiency. OF12-GEL achieved 41% release at 24 h and increased skin deposition to 323.7 μg/cm^2^, nearly 3-fold higher than free 5-FU (114.1 μg/cm^2^). In A431 cells, OF12 showed a lower IC₅₀ (0.77 μg/mL) than free 5-FU (1.15 μg/mL) and enhanced cellular uptake. *In vivo*, OF12-GEL significantly suppressed tumor growth in both DMBA-induced SCC rats and A431 xenograft mice, reducing tumor volume by and improving survival to 60%, and markedly downregulating BCL-2, Ki67, TNF-α, and ABCB1. OF12-GEL was non-irritant (PII = 0.33). These findings demonstrate a potent, safe, and targeted nanocarrier-based topical therapy for cSCC.

## Graphical abstract

**Figure:**
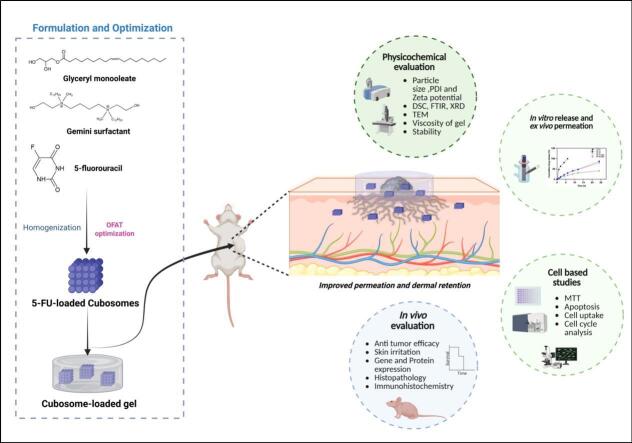

## 1 Introduction

Cutaneous squamous cell carcinoma (cSCC) is the second most common non-melanoma skin cancer, constituting approximately 20% of all skin malignancies. Globally, the incidence is at around 5 cases per 100,000 individuals, with a lifetime risk estimated at 14–20%, and continues to rise annually (Anderson et al., 2025). This growing burden is largely attributed to chronic ultraviolet radiation exposure, aging populations, and improved diagnostic capabilities. While early-stage cSCC is typically managed through surgical excision, cryotherapy, or photodynamic therapy, these interventions can be invasive, cosmetically disfiguring, or unsuitable for recurrent or diffuse lesions (Sharma et al., 2024). In such cases, topical chemotherapy emerges as a favorable alternative due to its non-invasive nature and potential for localized treatment.

Among topical chemotherapeutic agents, 5-fluorouracil (5-FU) remains a mainstay for the treatment of cutaneous malignancies, including basal cell carcinoma and squamous cell carcinoma (Gross et al., 2007; Neale et al., 2022). Despite its clinical utility, the therapeutic use of 5-FU is hampered by its low skin permeability and potential for systemic absorption, leading to adverse effects (Nikam et al., 2023). Therefore, significant efforts must be directed toward the development of next-generation topical formulations that enhance drug retention within the skin while minimizing systemic exposure. Over the years, Multiple approaches have been investigated to improve the permeability for 5-FU across the stratum corneum. Approaches such as chemical permeation enhancers, physical methods, and advanced nanoparticle-based systems have shown varying degrees of success (Tambunlertchai et al., 2021). Among these, nanocarriers, including liposomes, transfersomes, and cubosomes, have been in focus due to their ability to improve drug permeation, enhance physicochemical stability, and provide controlled, localized drug release within the skin (Gowda et al., 2023).

Cubosomes, lipid-based nanoparticles formed by self-assembly of amphiphilic lipids into a cubic phase stabilized by polymers, offer unique advantages for topical drug delivery due to their highly organized structure, large surface area, and ability to encapsulate hydrophilic, hydrophobic, and amphiphilic drugs (Abourehab et al., 2022). The physicochemical properties of cubosomes can be modulated to optimize skin permeation and drug release profiles (Janakiraman et al., 2019a). Their bicontinuous aqueous channels provide a much larger hydrophilic domain than liposomes or niosomes, enabling superior loading and sustained release of water-soluble drugs such as 5-FU. GMO-based cubosomes also demonstrate stronger skin bioadhesion, higher mechanical stability, and reduced leakage, making them particularly suitable for localized cutaneous drug delivery**.** These properties contribute to improved dermal deposition and reduced systemic exposure compared to lamellar vesicular systems.

Additionally, incorporating surfactants as permeation enhancers can further improve drug delivery efficiency. Among these, gemini surfactants exhibit superior interfacial activity and reduced toxicity compared to their monomeric counterparts, due to their lower critical micelle concentration and enhanced surface tension reduction (Ahmady et al., 2022). The gemini surfactants synthesized in this study were structurally tuned (symmetric dimeric headgroups, short spacer, and variable alkyl chain length) to provide enhanced electrostatic stabilization, improved packing at the lipid–water interface, and mild, reversible perturbation of the stratum corneum. These structural features differentiate them from commonly used commercial surfactants and previously reported gemini analogues, offering a more controlled enhancement of topical permeation.

For topical application, gels provide ease of use, prolonged skin contact, and improved patient compliance. Hydroxypropyl methylcellulose (HPMC) was selected as the gel-forming polymer due to its biocompatibility, non-ionic nature, and excellent film-forming properties. HPMC ensures uniform dispersion of cubosomes, stabilizes the formulation, and provides sustained drug release by increasing viscosity (Pan et al., 2023). Previous studies have demonstrated that HPMC-based hydrogels improve drug residence time, control release of actives, and are compatible with both hydrophilic and hydrophobic drugs, including nanocarrier-embedded systems for topical delivery (Amanzholkyzy et al., 2025; Rani et al., 2025). This evidence supports the rationale for using HPMC as the gel matrix in the present cubosome-based 5-FU formulation.

Herein, OF12-GEL was systematically developed and optimized using a One-Factor-At-a-Time (OFAT) approach, followed by comprehensive physicochemical characterizations, which ensured formulation stability and optimal performance. Furthermore, rigorous preclinical validation through *in vitro* and *in vivo* studies elucidated the therapeutic safety and efficacy of optimized OF12-GEL. Collectively, our findings emphasize the potential of this optimized novel formulation as a transformative topical nanotherapeutic platform for effective and safe management of cutaneous malignancies.

## 2 Materials and methods

### 2.1 Materials

Fluorouracil, 1-Bromododecane,1-Bromohexadecane, 1,4-Dibromobutane, 2-Methylaminoethanol,5-Bromouracil, 7,12 Dimethyl benz[*a*]anthracene and FITC (Fluorescein-5-isothiocyanate) were sourced from Sigma Aldrich Inc., USA. Glyceryl monooleate was procured from Mohini Organics Pvt. Ltd., Mumbai. Hydroxypropyl methylcellulose (HPMC K15M) was procured form Colorcon, Goa, India. MTT and Fetal Bovine Serum was sourced from HiMedia Laboratories Pvt. Ltd., Mumbai. DMEM-high glucose was sourced from Gibco-Thermo Fisher Scientific. All the reagents and solvents employed in the study were of analytical or HPLC grade.

### 2.2 Synthesis and characterization of Gemini surfactants

#### 2.2.1 Synthesis of Gemini surfactants

Two Gemini surfactants, *N*^1^,*N*^4^-didodecyl-*N*^1^,*N*^4^-bis(2-hydroxyethyl)-*N*^1^,*N*^4^-dimethylbutane-1,4-diaminium (GS12) and *N*^1^,*N*^4^-dihexadecyl-*N*^1^,*N*^4^-bis(2-hydroxyethyl)-*N*^1^,*N*^4^-dimethylbutane-1,4-diaminium (GS16) were synthesized based on a modified procedure from previous literature. Both compounds share a 4‑carbon spacer but differ in hydrophobic chain lengths—C12 for GS12 and C16 for GS16. The detailed synthetic routes are presented in Supplementary Information (Section: S1 and Fig. S1).

#### 2.2.2 Characterization of Gemini surfactants

The synthesized Gemini surfactants (GS12 and GS16) were subjected to various physicochemical characterization techniques. Critical micelle concentration (CMC) was assessed by measuring conductivity using a conductivity meter (Accumet XL 600D, Fisher Scientific, USA) equipped with a cell constant of 0.9 cm^−1^. Surfactant solutions were prepared in MilliQ water, and analysis was conducted 25 ± 2 °C. The instrument was calibrated using 0.1 M and 0.01 M KCl solutions, and the results represent the average of three independent readings (Talat et al., 2019). Mass spectrometry was performed using an Agilent ESI-LC/MS system coupled with a QTOF detector (Agilent 6545 QTOF LC/MS, USA) to identify the molecular ion peaks of the synthesized compounds. Structural confirmation was further supported by ^1^H and ^13^C NMR spectra recorded on a 500 MHz Bruker solid-state spectrometer (Bruker, Germany) using deuterated DMSO as the solvent. FTIR spectroscopy (Alpha II, Bruker, Germany) was conducted to identify functional groups. Differential Scanning Calorimetry (DSC-60, Shimadzu, Japan) was executed to assess thermal behavior. Additionally, crystallinity was assessed by recording XRD patterns using an X-ray diffractometer (Rigaku Ultima IV, Japan).

### 2.3 Preparation of 5-FU-loaded cubosomes

Prior to formulation, compatibility studies between 5-FU, glyceryl monooleate and synthesized Gemini surfactants were assessed using FTIR and DSC to ensure the absence of potential interactions. Cubosomes formulated employing the top-down technique, a widely adopted method for producing stable lipid-based nanoparticles. Initially, precise quantities of GMO and synthesized dimeric surfactants were accurately weighed and combined. This lipid-surfactant mixture was melted in a water bath maintained at 80 °C to ensure complete homogenization. Separately, 5-FU was solubilized in the aqueous phase. The molten lipid-surfactant blend was gradually introduced into the pre-heated aqueous phase under continuous high-speed homogenization (T25 digital ULTRA-TURRAX®, IKA, Germany) operating at 15,000 rpm for 5 min (Aboud et al., 2018). This process facilitated the formation of a uniform cubosomal dispersion. The resulting formulations were subsequently characterized for particle size, polydispersity index (PDI), zeta potential, and entrapment efficiency.

### 2.4 Optimization of 5-FU-loaded cubosomes

A systematic OFAT approach was adopted to optimize cubosomes by evaluating the individual effects of formulation variables on particle size, PDI, and zeta potential, while keeping other parameters constant. Two optimized formulations were developed for each of the synthesized Gemini surfactants. Initially, various lipid-to-Gemini surfactant ratios (9.5:0.5, 9:1, and 8:2) were screened, maintaining a total weight of 500 mg. Each formulation included 20 mg of 5-FU and was homogenized at 10,000 rpm for 5 min. Among the tested ratios, a 9:1 lipid-to-surfactant ratio yielded optimal physicochemical properties (Gaballa et al., 2020).

Subsequently, this optimized ratio (9:1) was fixed, and the amount of 5-FU was varied from 10 to 75 mg. Homogenization was maintained at 10,000 rpm for 5 min. Maximum drug entrapment efficiency was achieved with 30 mg of drug (lipid/ surfactant: drug ratio of 16:1). Based on these findings, both the lipid-to-surfactant ratio (9:1; total 500 mg) and drug amount (30 mg) were fixed for further optimization. Later, homogenization conditions were optimized by varying the duration (2.5, 5, and 7.5 min) and speed (8000, 10,000, and 12,000 rpm). The composition of various formulations is outlined in Table S1. Among them, formulations F26 and F36 demonstrated optimal characteristics and were henceforth designated as OF12 and OF16, respectively.

### 2.5 Preparation of cubosomal gel

The gel was prepared using the optimized concentration of HPMC, selected based on multiple trials evaluating parameters such as consistency, viscosity, and spreadability. To formulate the gel base, the required amount of HPMC was soaked overnight in distilled water at room temperature to ensure complete hydration. Methylparaben (0.18% *w*/w) and propylparaben (0.02% w/w) were employed as preservatives, while propylene glycol (2% *w*/*v*) was incorporated as a cosolvent to enhance spreadability. The composition of the prepared gels (10 g batch size) is provided in Table S2). The optimized formulations OF12_G2 and OF16_G2 were designated as OF12-GEL and OF16-GEL, respectively, for subsequent evaluations. For drug-loaded formulations, cubosomal dispersions equivalent to 1% w/w of 5-FU were incorporated into the HPMC gel base by thorough mixing to ensure homogeneity. The final gels were held at 4 °C in amber‑tinted, airtight containers to protect from light and degradation

### 2.6 Characterization of cubosomes

The particle size, PDI, and zeta potential of the developed cubosomal dispersions (OF12 and OF16) were measured using a Zetasizer (Nano Series 3600, Malvern Instruments, UK). Entrapment efficiency (EE%) was determined by ultracentrifuging the dispersions at 22,000 rpm for 30 min. The resulting pellet was redispersed in water, treated with 2% Triton X-100 in methanol, and sonicated to ensure complete lysis. Post centrifugation (10,000 rpm, 10 min), the supernatant was collected and examined using RP-HPLC. EE% was determined using the following equation

$$\mathrm{EE}\%=\frac{\mathrm{Amount of entrapped drug}}{\mathrm{Total amount of drug added}}\;X\;100$$

To improve the stability, the optimized formulations were freeze-dried using mannitol, trehalose, and sucrose as cryoprotectants at varying concentrations (2.5%, 5%, and 10%). The dispersions were held at −80 °C for 24 h and Freeze-drying was carried out at −60 °C under a vacuum of <0.1 mbar for 48 h. The resulting powders were evaluated for flowability, particle size, and PDI. Based on retention of physicochemical characteristics, 5% mannitol was selected as the optimal cryoprotectant (Shi et al., 2017).

Further characterization of the lyophilized cubosomes included FTIR spectroscopy to assess component interactions, DSC for thermal behavior, and XRD to analyze the crystalline nature of the formulations. Morphological examination was performed using TEM (Tecnai G2 Spirit Biotwin, FEI Company, USA), wherein diluted sample droplet was added on a carbon-coated copper grid, negatively stained with phosphotungstic acid, dried, and imaged (Shreya et al., 2016).

### 2.7 Characterization of cubosome-loaded gel

The appearance, color, and odour of the prepared gels were evaluated subjectively. The pH was measured using a digital pH meter. Viscosity was determined at room temperature using a viscometer (spindles SP29 and L4) operated at speeds ranging from 2 to 20 rpm. To assess spreadability, 1 g of the gel was sandwiched between two glass slides and subjected to a 200 g weight for 1 min, after which the spread area was measured used to calculate spreadability as per previously reported method (Gollavilli et al., 2020).

$$\text{Spreadability}=\frac{\mathrm{weight}\;x\;\mathrm{Area of}\;\mathrm{gel}\;\mathrm{spread}\;\mathrm{on}\;\mathrm{the slide}}{\mathrm{time}\;}$$

### 2.8 *In vitro* drug release profiling

5-FU release from the optimized formulations OF12 and OF16 and free 5-FU solution was assessed using vertical-type diffusion cell, with slight modifications to previously reported method (Charankumar et al., 2023). A dialysis membrane (molecular weight cut-off: 12 kDa) was positioned between the donor and receptor chambers. The receptor chamber was filled with phosphate buffer (pH 7.4) (3.5 mL) and held at 37 ± 0.5 °C under continuous stirring. The donor compartment was loaded with either 5-FU solution (10 mg/mL) or the respective formulations incorporating an equivalent drug dose. At fixed time points, 0.35 mL was sampled from the receptor chamber, and an equal amount of fresh buffer was introduced to uphold sink conditions. The amount of 5-FU present was quantified using HPLC. Additionally, to elucidate the drug release mechanism, the release profiles were fitted to various kinetic models (Raychaudhuri et al., 2021).

### 2.9 Stability study

The stability of optimized formulations were determined at of 4 ± 3 °C and 25 ± 2 °C/60 ± 5% relative humidity for 90 days (Vambhurkar et al., 2024). To monitor stability over time, particle size, PDI, and ZP were assessed at 15-day intervals up to 90 days.

### 2.10 *In vitro* cell culture studies

#### 2.10.1 Cell viability assay

MTT assay was employed to evaluated cell viability. Briefly, A431 and HaCaT cells were cultured in DMEM (high glucose) supplemented with 10% FBS and 1% antibiotic-antimycotic under 5% CO₂ and 18–20% O₂ at 37 °C. 2 × 10^4^ cells/well were plated in 96-well plates and cultured for 24 h, subsequently administered with various concentrations of test agents for another 24 h. After aspirating the medium, MTT solution (0.5 mg/mL) was added to each well and incubated for 3 h. The obtained formazan crystals were solubilized in DMSO, and the absorbance was recorded at 570 nm using a plate reader (BioTek Instruments Inc., US) (Pandey et al., 2020). % Cell viability was then determined using the formula given below.

$$\%\text{Cell viability}=\frac{\text{Absorbance of treated cells}}{\text{Absorbance of untreated cells}}X\;100$$

#### 2.10.2 Cellular uptake studies

To access the cellular internalization of prepared nanovesicles, A431 cells were plated in a 6-well plate at 2 × 10^5^ cells/ well density and were cultured for 24 h at 37 °C with CO₂. Later, the cells were treated with FITC-labelled unconjugated and conjugated formulations or FITC solution as a control. Following 6 h incubation, cells were rinsed thrice with PBS and trypsinized, and cells were re-dispersed in PBS and placed in FACS tube for analysis using flow cytometry (BD CellQuest Pro v6.0) using a 488 nm excitation laser and 535 nm (FL1) for detection (Yuan et al., 2016).

#### 2.10.3 Cell cycle analysis

A431 cells (2 × 10^5^/well) were plated in 6-well plates and maintained at 37 °C with 5% CO₂ for 24 h After treatment with IC₅₀ concentrations of optimized cubosomes or pure 5-FU for 24 h, subsequently the cells were rinsed with PBS and then detached by treating with 250 μL of trypsin-EDTA solution. Following centrifugation (300 ×*g*, 5 min, 25 °C) and PBS wash, cells were fixed in cold 70% ethanol (added dropwise) and stored at −20 °C for 30 min. After washing, cells were exposed to 400 μL PI/RNase buffer, incubated in the dark for 15–20 min at room temperature, the samples were analyzed by flow cytometry (Jain et al., 2025).

#### 2.10.4 *Ex vivo* skin permeation

Vertical diffusion cells (diffusion area: 1 cm^2^) were employed to assess the *ex vivo* skin permeation. Excised dorsal skin from Wistar rats was sandwiched between the donor and receptor chambers with the epidermis facing the donor side. Both compartments were filled with PBS (pH 7.4) and equilibrated at 37 ± 0.5 °C for 45 min under continuous stirring. Following equilibration, the donor chamber was loaded with either 5-FU solution (10 mg/mL) or optimized formulations (OF12/OF16) containing an equivalent amount of drug. At specific intervals (1, 2, 4, 6, 9, 12, and 24 h), 0.35 mL aliquots were sampled from the receptor chamber and replenished with fresh media. Drug content was quantified using HPLC. Post 24 h, the skin was washed, minced, and homogenized in PBS. The homogenate was centrifuged, filtered (0.45 μm), and analyzed by a previously developed HPLC method (Mutalik et al., 2014).

### 2.11 *In vivo* safety and efficacy

#### 2.11.1 Skin irritation studies

The study used male Wistar rats aged 6–8 weeks (200–250 g), housed in polypropylene cages under controlled temperature (24–26 °C) and a 12-h light/dark schedule, with unlimited access to food and water. The protocol for animal experiments was approved by the Institutional Animal Ethical Committee, Kasturba Medical College, Manipal Academy of Higher Education, Manipal (IAEC/KMC/112/2020) and (IAEC/KMC/94/2022). The rats were segregated into four groups: untreated control, formalin (0.8% *v*/v) treated, placebo formulation treated, and OF12-GEL treated (100 mg gel containing 1% 5-FU). The formulations were applied once daily to the shaved area (∼5 cm^2^) for seven consecutive days. Throughout the study, the skin was monitored regularly for any morphological changes. Post seven days, the skin was visually inspected for signs of edema and erythema and were scored accordingly. The Primary Irritation Index (PII) was calculated, and formulations were categorized as non-irritating, slightly irritating, moderately irritating, or severely irritating as outlined in Table S3. Following the assessment, animals were euthanized, and the skin tissues were excised for histopathological evaluation (Joshi et al., 2018).

$$\mathrm{Primary irritation index}\left(\mathrm{PII}\right)=\frac{\mathrm{Score for erythema}+\mathrm{Score for edema}}{\mathrm{Total score}}$$

#### 2.11.2 FTIR spectroscopic analysis of the stratum corneum

To evaluate the impact of Gemini surfactants on stratum corneum (SC) lipids, FTIR analysis was conducted on SC samples treated with or without Gemini surfactants, following a previously reported protocol. Briefly, SC was isolated from full-thickness skin by overnight incubation in a 0.5% sodium bicarbonate solution containing 0.1% trypsin (Banas et al., 2024). After separation, the SC layers were thoroughly dried. Small sections of the dried SC were then incubated with either PBS (control) or a Gemini surfactant solution (10 mg/mL) for 2 h. Following incubation, the solutions were removed by gentle wiping, and the SC samples were dried and analyzed using FTIR (Alpha II, Bruker, Germany).

#### 2.11.3 *In vivo* antitumor efficacy

The *in vivo* anticancer efficacy of the optimized cubosomal gel (OF12-GEL; containing 1% *w*/w 5-FU) was evaluated using two animal models: a chemically induced squamous cell carcinoma (SCC) model in Wistar rats and an A431 xenograft tumor model in nude mice. The marketed formulation (Flonida cream 1% *w*/w) was used as a reference control in both models.

##### 2.11.3.1 Squamous cell carcinoma model

SCC was induced in Wistar rats by topical application of 200 μL of freshly prepared 1% *w*/*v* 7,12-dimethyl-benzanthracene (DMBA) in acetone on the shaved dorsal skin, followed by exposure to UV light (311 nm) for 1 min. This procedure was repeated on alternate days for 3 months (Rajmani et al., 2011). The development of SCC was confirmed by histological analysis of skin samples preserved in 10% formalin. After induction, rats were segragated into four groups (Normal control (normal animals with no treatment), Positive control (disease induced animals with no treatment), Marketed formulation (disease induced treated with Flonida cream containing 1% w/w of 5-FU), and Optimized formulation (disease induced treated with OF12-GEL containing 1% w/w of 5-FU)) with 6 animals per group. Treatments were applied topically once daily for 30 days. Visual observations were made throughout the study to monitor morphological changes. Post treatment, rats were sacrificed, and skin samples were collected for histopathological evaluation following hematoxylin and eosin (H&E) staining.

##### 2.11.3.2 A431 xenograft tumor model

Female athymic nude mice (Crl:NU(NCr)-Foxn1nu), weighing 18–20 g and aged 4–5 weeks, were employed. The animals were habituated for one week under sterile conditions, maintained at 20–24 °C and relative humidity of 30–70%, with a 12-h light/dark cycle. Throughout the study, the nude mice were fed with an autoclaved diet and reverse osmosis water *ad libitum*.

A431 cells (5 × 10^6^ per site, suspended in a 1:1 mixture with matrigel) were inoculated subcutaneously into both flanks of the nude mice (Shao et al., 2006) and allowed for the tumor development. After tumors reached approximately animals were randomized (*n* = 6) into three groups (namely, disease control, Marketed formulation and OF12-GEL). Tumor progression was regularly assessed, and upon reaching a volume of ∼150 mm^3^, the animals were randamized into three groups (based on tumor volume on day 6), Disease control, Marketed formulation (Flonida cream 1% *w*/w), and Optimized cubosomal gel (OF12-GEL; 100 mg gel containing 1% 5-FU)). The respective formulations were applied topically to the tumor site (∼1 cm × 2 cm) every day from day 7 to day 21 s. Body weight and tumor dimensions were monitored every other day. Tumor volume was determined using the following equation.

Tumor Volume (V) = 0.5 x a x b2.

Tumor Area was determined using the following equation: 4π x a x b.

where V is tumor volume, ‘a’ is the long diameter of the tumor, and ‘b’ is the short diameter of the tumor.

Post study, tumors were excised and processed for molecular and histological analyses. Portions of tumors were flash-frozen for RNA and protein extraction, while others were fixed in 10% neutral buffered formalin. The animal experimental protocol, pertaining to the preclinical *in vivo* studies explained in this section, was approved Institutional Animal Ethics Committee (IICT/IAEC/055/2022) of CSIR-Indian Institute of Chemical Technology (CSIR-IICT), Government of India, Hyderabad.

##### 2.11.3.3 Gene and protein expression analysis

###### 2.11.3.3.1 Quantitative real-time PCR (RT-qPCR)

Total RNA was extracted from tumor tissues using RNAiso Plus reagent, followed by extraction *via* the Trizol–chloroform method, as per the reported protocol (Andugulapati et al., 2020). Subsequently, RNA concentration and purity were assessed using a NanoDrop and 1 μg of total RNA was reverse transcribed into complementary DNA using a commercial cDNA synthesis kit. Gene-specific primers for *BAX* and *BCL2* were designed using Primer3 software and synthesized by Sahagene Limited (Hyderabad, India). Normalization was performed using *β2-microglobulin* as the reference gene. RT-qPCR was conducted using SYBR Green Master Mix on Real-Time PCR system. The amplification conditions followed the standard protocol provided with the SYBR Green kit. Gene expression was determined using the 2^−ΔΔCt method and reported as mean ± standard error of the mean

###### 2.11.3.3.2 Western blot analysis

BCL-2 expression was evaluated by Western blotting. Briefly, tumor specimens were homogenized in RIPA lysis buffer, and protein was quantified using BCA assay. Samples containing 30 μg of protein each were loaded onto 10–12% Bis-Tris SDS-PAGE gels for separation, followed by transfer onto PVDF membranes. Following blocking the membranes with 5% BSA at room temperature for 1 h, they were incubated overnight at 4 °C with primary antibodies targeting BCL-2. Subsequently, membranes were exposed to secondary antibodies for 1 h at room temperature. Protein bands were detected using an enhanced chemiluminescence detection system and captured with the Bio-Rad ChemiDoc™ Touch Imaging System (Tirunavalli et al., 2021). Densitometric analysis was performed using ImageJ software, with band intensities normalized to β-actin and expressed as mean ± standard error of the mean.

### 2.12 Statistical analysis

The data were statistically assessed with the aid of GraphPad Prism software, version 8.0.1 (GraphPad Software Inc., USA). One-way ANOVA succeeded by Dunnet's test (for comparison of the results with control) or Tukey's test (for comparison of multiple groups). Values of *p*< 0.05 were taken to be statistically significant.

## 3 Results and discussion

### 3.1 Synthesis and characterization of Gemini surfactants

**Fig. 1:**
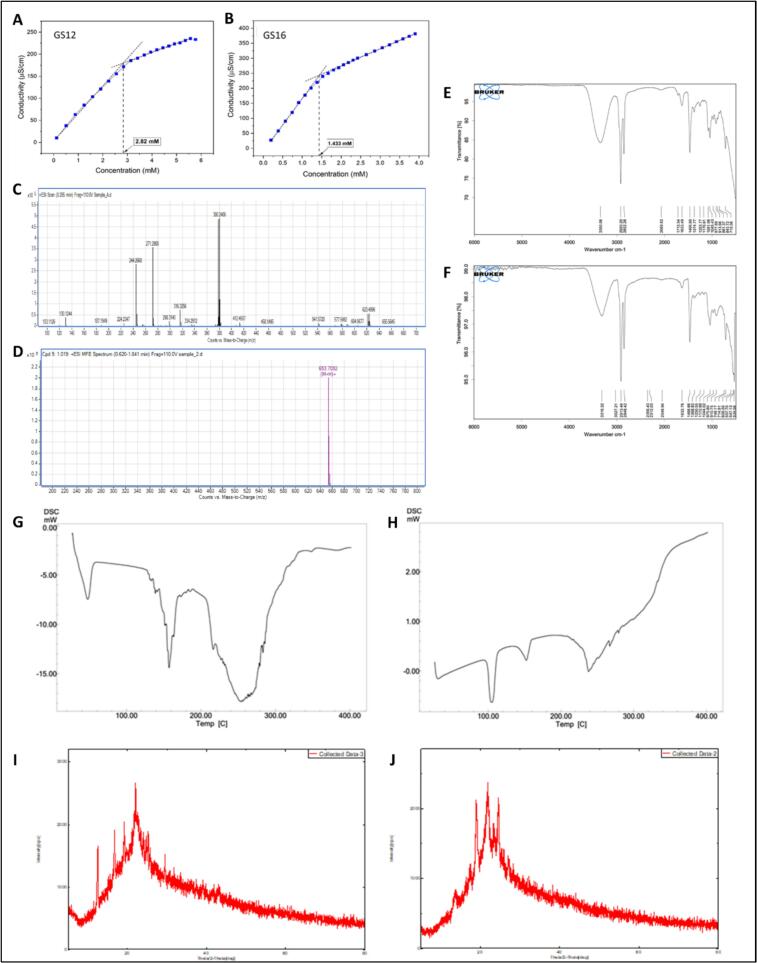
Physicochemical characterization of gemini surfactants GS12 and GS16. (A, B) Conductivity *versus* concentration plots for critical micelle concentration (CMC) determination of GS12 and GS16, respectively. (C, D) Mass spectra of GS12 and GS16 dimeric surfactants. (E, F) FTIR spectra of GS12 and GS16. (G, H) DSC thermograms of GS12 and GS16. (I, J) X-ray diffraction patterns of GS12 and GS16, respectively.

Two Gemini surfactants, N1,N4-didodecyl-N1,N4-bis(2-hydroxyethyl)-N1,N4-dimethylbutane-1,4-diaminium (GS12) and N1,N4-dihexadecyl-N1,N4-bis(2-hydroxyethyl)-N1,N4-dimethylbutane-1,4-diaminium (GS16), differing in alkyl chain length (C12 and C16 respectively), were successfully synthesized and characterized. Electrical conductivity measurements determined their critical micelle concentrations (CMCs) as 2.82 mM for GS12 and 1.433 mM for GS16 **(**Fig. 1A and B**)**, which are significantly lower than conventional surfactants such as sodium dodecyl sulfate (8.2 mM), dodecylbromide (7.5 mM), and hexadecylbromide (5.5 mM), indicating efficient micellization (Bahr et al., 2019). HLB values were calculated as 7.5 and 6.2 for GS12 and GS16, respectively. The yield for GS12 and GS16 was 76% and 78% respectively. Mass spectrometry analysis revealed molecular ion peaks at *m*/*z* 541.5 for GS12 and 653.7 for GS16, confirming their expected molecular weights **(**Fig. 1C and D**)**. However, GS12 exhibited low molecular ion peak intensity due to fragmentation, likely caused by its quaternary nitrogen atoms and long alkyl chains. The most prominent fragment peak at m/z 380 resulted from cleavage of the alkyl chains, accompanied by several smaller fragment ions. Additionally, pseudo molecular ion peaks appeared adjacent to the molecular ions, attributed to the recombination of fragments with the parent molecule. These results confirm the successful synthesis of the Gemini surfactants, despite fragmentation during the detection process. ^1^H and ^13^C NMR spectroscopy further confirmed the molecular structures of both surfactants (Fig. S2, Table S4–7). FTIR analysis showed characteristic absorption peaks at 719 cm^−1^ (CH_2_ rocking), 1045 cm^−1^ (C–N^+^ bond), and 3350 cm^−1^ (OH stretching), with CH symmetric bending at 1374 cm^−1^, CH symmetric stretching at 2852 cm^−1^, and CH asymmetric stretching at 2920 cm^−1^ **(**Fig. 1E and F**)**, consistent with literature (Aiad et al., 2016; Khalaf et al., 2017).

Differential Scanning Calorimetry (DSC) thermograms **(**Fig. 1G and H**)** displayed endothermic peaks for GS12 at 47.94 °C, 157.34 °C, and 254.06 °C, and for GS16 at 105.23 °C, 152.39 °C, and 238 °C, indicative of polymorphic and phase transitions associated with their hydrophobic and ionic domains, in agreement with previously reported data (Jurasin et al., 2011). X-ray diffraction (XRD) patterns showed multiple sharp diffraction peaks **(**Fig. 1I and J**)**, confirming the crystalline nature of the quaternary ammonium salts formed (He et al., 2019). Overall, these results confirm the successful synthesis, expected molecular structures, and physicochemical stability of GS12 and GS16 Gemini surfactants, supporting their suitability for formulation applications.

### 3.2 Formulation and optimization of cubosomes

The cubosomal formulations containing lipid monoolein, synthesized Gemini surfactants (GS12 or GS16), and 5-fluorouracil (5-FU) were systematically optimized OFAT approach, and the results of drug excipient compatibility studies are presented in the Supplementary Information (Section S2 and Fig. S3). The primary aim was to achieve cubosomes with high drug %EE, minimal particle size, and low PDI, key factors for effective topical delivery. Initially, the lipid-to-surfactant ratio was varied (9.5:0.5, 9:1, and 8:2; total lipid and surfactant fixed at 500 mg), with 20 mg drug per batch, the ratios were selected based on previous literature (Elgindy et al., 2016; Gaballa et al., 2020). Both GS12 and GS16 surfactants were evaluated separately. The 9:1 ratio (formulations F2 and F5) yielded the smallest particle sizes, optimal PDI, and highest %EE, thus selected for further optimization. Next, with the 9:1 ratio fixed, drug loading was varied from 10 to 75 mg. Entrapment efficiency peaked at around 30 mg drug addition, achieving ∼59% for GS12 (F9) and 60% for GS16 (F15). Drug amounts below or above 30 mg resulted in lower %EE values, highlighting 30 mg as the optimal loading.

**Fig. 2:**
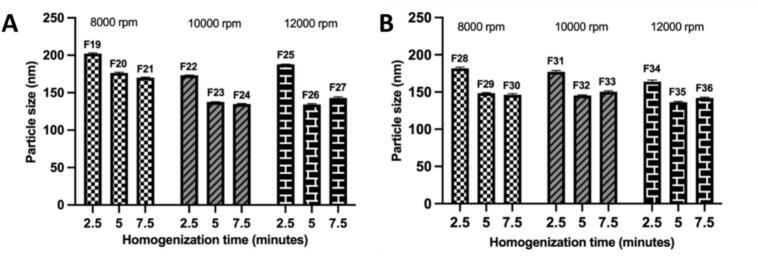
Effect of high-shear homogenization speed and time on particle size of A) GS12 cubosomes and B) GS16 cubosomes. Formulation codes F19–F36 denote the specific cubosomal batches prepared under different homogenization conditions (Tables S8 and S9), where F19–F27 correspond to GS12 formulations and F28–F36 correspond to GS16 formulations.

Subsequent optimization focused on high-speed homogenization (HSH) parameters. Speed (8000–12,000 rpm) and time (2.5–7.5 min) were varied (F19-F36) (Table S8–9). Increasing HSH speed and duration consistently reduced particle size, attributed to enhanced shear forces breaking down the cubic gel into nanosized vesicles (Abdel-Bar and el Basset Sanad, 2017). For GS12 cubosomes, the smallest size (134.4 nm) was observed at 12000 rpm for 5 min (F26), while GS16 cubosomes showed optimal size (137.5 nm) at the same speed and time (F35). Extending homogenization to 7.5 min slightly increased particle size but improved entrapment efficiency by 2% (GS12, F27) and nearly 10% (GS16, F36) **(**Fig. 2**)**. Considering this trade-off, F26 and F36 were chosen as optimized formulations.

The optimized cubosomes, designated OF12 (GS12) and OF16 (GS16), featured particle sizes between 134 and 141 nm, PDI values below 0.26, and highly positive zeta potentials (+69.3 mV and +57 mV) **(**Table 1**)**, indicating excellent colloidal stability and homogeneity. Particle sizes under 300 nm are favorable for deep skin penetration, supporting their potential for topical application. The findings align well with previous reports where monoolein-to-surfactant ratios around 9:1 yielded similarly small particle sizes and stable dispersions (Gaballa et al., 2020). The high positive zeta potentials suggest strong electrostatic repulsion, preventing aggregation, crucial for maintaining formulation stability (Clogston and Patri, 2011).

**Table 1:** Particle size, PDI, zeta potential and % EE for variation in lipid to surfactant ratio (*n*=3).

Formulation code	Surfactant	Ratio (Lipid: Surfactant)	Particle size (nm)	PDI	Zeta potential (mV)	% EE of 5-FU
F1	GS12	9.5:0.5	143.7±1.09	0.206±0.01	+66.6±1.4	49.3±0.58
F2		9:1	134.4±2.19	0.231±0.07	+76.1±0.7	51.3±0.56
F3		8:2	170.5±2.36	0.384±0.01	+78.2±1.05	47.8±1.17
F4	GS16	9.5:0.5	150.1±1.84	0.136±0.02	+52.1±1.27	57.1±0.58
F5		9:1	145.5±1.56	0.160±0.05	+49.1±0.55	65.1±1.92
F6		8:2	161.4±3.19	0.343±0.06	+59.4±1.70	55.2±1.47

Overall, the systematic optimization underscored the critical influence of surfactant chain length, lipid-surfactant ratio, drug loading, and homogenization parameters on the cubosomal characteristics, ultimately enabling the development of stable, nanosized cubosomes with efficient 5-FU loading for topical delivery.

### 3.3 Characterization of cubosomes

Optimized cubosomal dispersions (OF12 and OF16) were lyophilized using various cryoprotectants to retain their physicochemical characteristics post-drying. Among all tested options, 5% mannitol provided the most favorable outcome, with only a slight increase in particle size and PDI values as shown in Table S10. Other cryoprotectants such as trehalose and sucrose, particularly at higher concentrations, led to substantial increases in particle size and polydispersity, indicating aggregation or structural disruption during freeze-drying. Therefore, 5% mannitol was chosen as the optimal cryoprotectant for further studies.

**Fig. 3:**
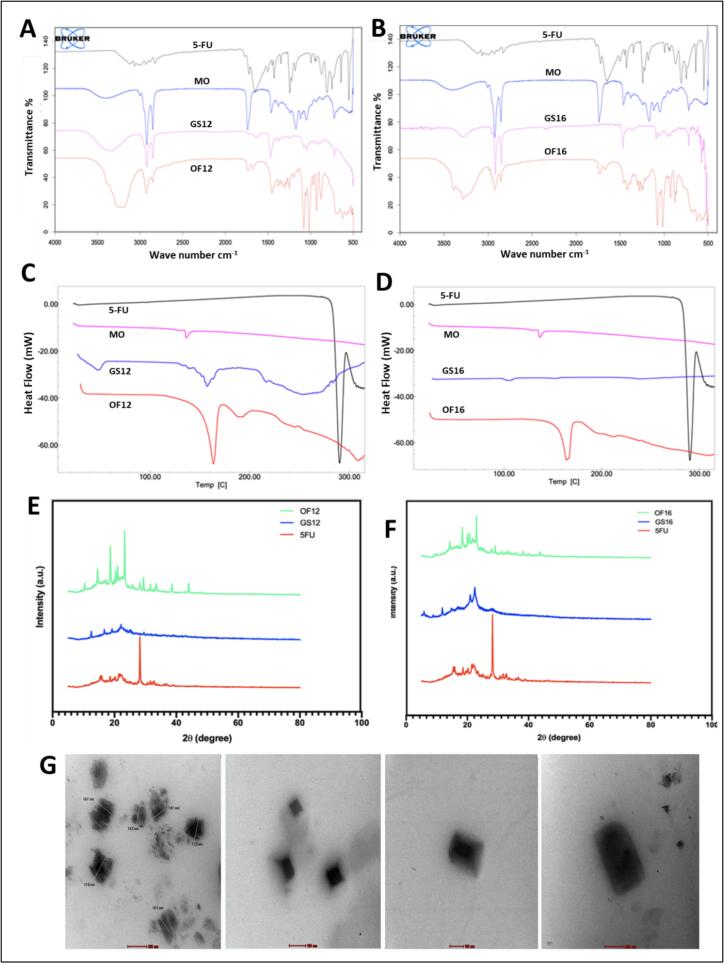
Characterization of optimized cubosomal formulations OF12 (GS12 system) and OF16 (GS16 system). FTIR spectra of (A) pure 5-FU, monoolein (MO), GS12, and OF12 cubosomes. (B) pure 5-FU, MO, GS16, and OF16 cubosomes. DSC thermograms of (C) pure 5-FU, MO, GS12, and OF12 cubosomes. (D) pure 5-FU, MO, GS16, and OF16 cubosomes. XRD patterns of (E) pure 5-FU, GS12, and OF12 cubosomes. (F) pure 5-FU, GS16, and OF16 cubosomes. (G) TEM images of optimized cubosomes: OF12 at 43,000×, 105,000×, and 87,000× magnifications (from left to right); OF16 at 43,000× magnification (rightmost).

FTIR analysis confirmed the successful molecular encapsulation of 5-FU within the cubosomal matrix, as evidenced by the absence or significant shifts in its characteristic peaks—C–F stretching at 1275 cm^−1^, C=O stretching at 1638 cm^−1^, and a broad N—H stretching band between 3000 and 3500 cm^−1^ in the spectra of OF12 and OF16 **(**Fig. 3A and B**)**. Monoolein exhibited its typical OH stretching at ∼3300 cm^−1^, CH stretching at 2920 and 2845 cm^−1^, and C=O stretching at 1728 cm^−1^ (Tang et al., 2008). The retention of characteristic peaks of monoolein and Gemini surfactants in the cubosomal formulations suggests the absence of chemical interaction among components.

Complementing the FTIR findings, DSC thermograms **(**Fig. 3C and D**)** provided further confirmation of 5-FU encapsulation. The sharp melting endotherm of crystalline 5-FU at 286 °C was absent in both OF12 and OF16, indicating drug amorphization upon entrapment. The appearance of an endothermic peak around 165 °C corresponded to the melting point of mannitol, used as a cryoprotectant during lyophilisation (Jaipal et al., 2015). Gemini surfactants retained their characteristic transitions, GS12 at 47.94 °C, 157.34 °C, and 254.06 °C, and GS16 at 105.23 °C, 152.39 °C, and 238 °C, indicating excipient stability. Corroborating these results, XRD analysis **(**Fig. 3E and F**)** revealed a transformation from the highly crystalline nature of pure 5-FU to an amorphous pattern in the optimized cubosomal formulations. The disappearance of sharp diffraction peaks and the presence of broad halos indicate a loss of long-range order, further supporting the efficient physical entrapment of 5-FU within the lipid matrix. Finally, TEM imaging demonstrated the morphological integrity of the Cubosomes **(**Fig. 3G**)**. The formulations exhibited discrete, non-aggregated particles with polyangular to cubic shapes, consistent with their bicontinuous internal structure (Nasr et al., 2020). Observed particle sizes were below 200 nm and closely matched the values obtained from DLS analysis, confirming uniform nanoscale dispersion and successful formulation.

### 3.4 Characterization of cubosomal based gel

To enable effective topical application, cubosomal gels containing 1% *w*/w 5-FU were developed using varying concentrations of HPMC. A clear concentration-dependent increase in gel viscosity was observed (Fig. S4 and Table S11), with cubosomal formulations consistently exhibiting higher viscosity than their corresponding blank gels, attributable to the presence of monoolein. Among the tested concentrations, gels formulated with 1.5% HPMC (designated as OF12-GEL and OF16-GEL) demonstrated optimal consistency and were selected for further evaluation. These gels exhibited desirable aesthetic and physicochemical attributes—white, homogenous, smooth in texture, and pH-adjusted (5.8 ± 0.3) to match the skin's physiological range, ensuring dermal compatibility without irritancy. Moreover, both gels demonstrated excellent spreadability, with values of 16.21 ± 0.34 (OF12-GEL) and 20.16 ± 0.18 (OF16-GEL), indicating ease of application and uniform skin coverage.

### 3.5 *In vitro* 5-FU release profile

**Fig. 4:**
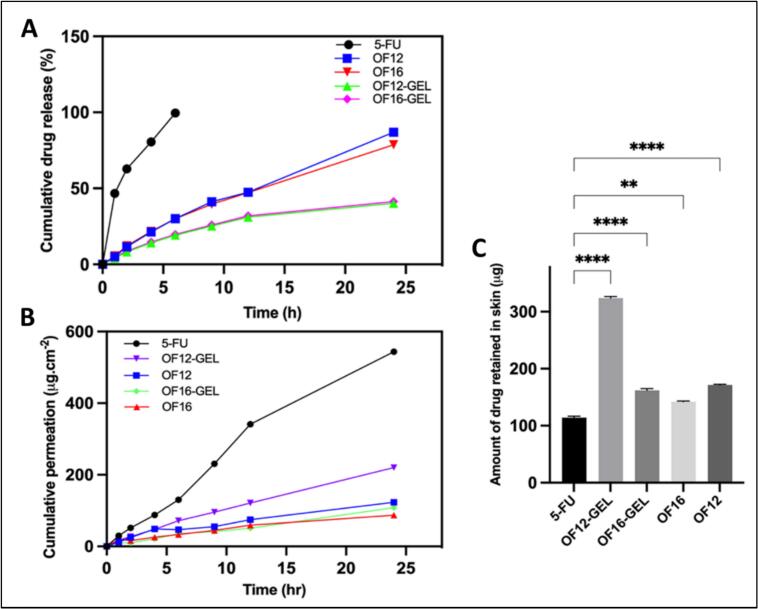
(A) *In vitro* 5-FU release profiles of free 5-FU solution, cubosomal formulations (OF12, OF16), and gel formulations (OF12-GEL, OF16-GEL). (B) *Ex vivo* skin permeation profiles of free 5-FU solution, optimized cubosomal formulations (OF12, OF16), and gel formulations (OF12-GEL, OF16-GEL). (C) Skin retention of free 5-FU solution, optimized cubosomal formulations (OF12, OF16), and gel formulations (OF12-GEL, OF16-GEL). Data represent mean ± SD (*n* = 3). Statistical significance compared to free 5-FU solution: **p < 0.001, ****p < 0.0001.

Free 5-FU solution exhibited a burst release, with approximately 75% of the 5-FU released within 12 h and reaching 93% at 24 h. In contrast, the optimized cubosomal dispersions (OF12 and OF16) showed a sustained release pattern, with about 47% release at 12 h and 79–87% by the end of 24 h. The gel formulations (OF12-GEL and OF16-GEL) demonstrated an even slower release, with only ∼30% of drug released at 12 h and 41% at 24 h **(**Fig. 4A**)**.

The above results suggest sustained release capability of cubosomal formulations, which could be ascribed to the encapsulation of 5-FU within their structured lipid matrix, which acts as a reservoir and controls diffusion. The additional viscosity of the gel matrix further retards drug diffusion, leading to the most prolonged release among the tested formulations. These findings align well with previous reports on the release-retarding properties of cubosomes and their ability to act as a dermal depot system (Rapalli et al., 2021). Furthermore, kinetic analysis revealed that the release profile of OF12 was best fitted to the Higuchi model (R^2^ = 0.989), demonstrating a biphasic release governed by diffusion, with an initial rapid release succeeded by a prolonged, sustained phase.Such behavior aligns with prior reports on cubosomes, supporting their potential for controlled topical drug delivery (Kurangi et al., 2021).

### 3.6 Stability study

The optimized cubosomal formulation (OF12) was stored at 5±3 °C and 25±2 °C/ 60±5% RH for 3 months and assessed for variation in particle size and zeta potential at regular intervals during this time period (Managuli et al., 2019). Table 2 illustrates particle size and zeta potential results for OF12 cubosomes upon storage for three months. Formulations showed slight increase in particle size (<5%) and zeta potential (<5%) when held at 5±3 °C. Upon storage at 25±2 °C, the variation in particle size and zeta potential was slightly more (<10%). The reason for the difference is mostly due to degradation of the formulation at these temperature and humidity conditions.

**Table 2:** Three month stability profile of OF12 formulation.

Number of days	Storage conditions	Particle size (nm)	Zeta potential (mV)
0	5±3 °C/ 60±5%	136.2±1.5	+70.2±1.32
30		138±1.22	+71±1.18
60		139±1.31	+71.7±2.21
90		142±1.8	+73.6±2.01
0	25±2 °C/ 60±5%	135.3±1.23	+69±1.2
30		140.2±1.6	+71.3±1.68
60		143.7±2.33	+73.4±1.9
90		147.8±2.41	+74.4±2.55

### 3.7 *Ex vivo* skin permeation and skin retention

The cubosomal formulations significantly enhanced skin retention of 5-FU compared to the free drug. Free 5-FU exhibited a high cumulative permeation of 543.54 ± 5.15 μg/cm^2^ over 24 h (Table S12, Fig. 4B), whereas all cubosomal systems showed substantially reduced permeation (*p* < 0.0001). Among these, OF12 and OF12-GEL outperformed OF16 counterparts, with permeation values of 123.33 ± 0.49 μg and 220.15 ± 0.5 μg, respectively.

Notably, OF12-GEL achieved the highest drug deposition within the skin (323.71 ± 4.25 μg/cm^2^), nearly threefold greater than free 5-FU (114.14 ± 3.41 μg/cm^2^), underscoring its superior localized delivery **(**Fig. 4C**).** The enhanced retention is attributed to the structural similarity of cubosomes to the stratum corneum and their depot-forming capacity. Moreover, the inclusion of monoolein and gemini surfactants synergistically disrupts stratum corneum lipid packing, enhancing permeation and drug localization. Collectively, these findings highlight the promise of gemini surfactant-integrated cubosomal gels as a potent, targeted platform for topical 5-FU delivery.

### 3.8 *In vitro* cell culture studies

#### 3.8.1 Cell viability assay

**Fig. 5:**
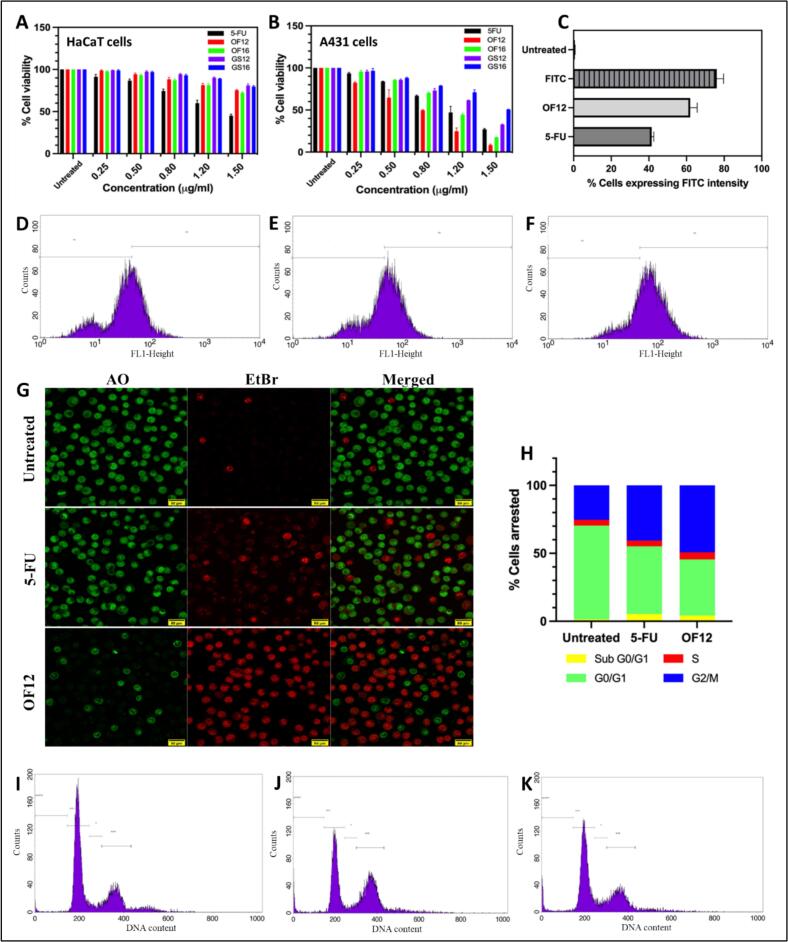
*In Vitro* Cellular Studies: Percentage cell viability of pure drug, optimized cubosomal formulations (OF12, OF16), and synthesized dimeric surfactants (GS12, GS16) in (A) HaCat and (B) A431 cell lines. (C) Cellular uptake profiles of FITC, OF12, and free 5-FU in A431 cells. Flow cytometry histograms of A431 cells treated with (D) 5-FU, (E) OF12, and (F) FITC; gated singlets distinguish M1 (negative) and M2 (positive) populations, with % M2 used for quantification. (G) Confocal microscopy images of AO/EB-stained A431 cells: untreated, 5-FU treated, and OF12 treated (green: viable cells; red: dead/damaged cells; 40× magnification). (H) Cell cycle phase distribution (%) of A431 cells following different treatments. Representative flow cytometry histograms of cell cycle phases in A431 cells (I) untreated control (J) 5-FU treated, and (K) OF12 treated. (For interpretation of the references to color in this figure legend, the reader is referred to the web version of this article.)

The cytotoxic potential of the developed cubosomal formulations was evaluated in HaCaT and A431 cell lines. A concentration-dependent decrease in cell viability was observed for all tested samples, including pure 5-FU, gemini surfactants (GS12, GS16), and optimized cubosomal formulations (OF12, OF16), as presented in Fig. 5A and B. The IC₅₀ values are summarized in Table S13 and Fig. S5. In HaCaT cells, the IC₅₀ of pure 5-FU was found to be 1.44 ± 0.07 μg/mL, while OF12 and OF16 exhibited higher IC₅₀ values of 3.03 ± 0.05 μg/mL and 2.82 ± 0.16 μg/mL, respectively. This suggests that cubosomal encapsulation of 5-FU significantly reduced its cytotoxicity in healthy cells. Additionally, Gemini surfactants GS12 and GS16 exhibited minimal cytotoxicity toward HaCaT cells, further confirming their biocompatibility. These findings align with previous studies reporting that cubosomes (Fahmy et al., 2021; Mehanna et al., 2020) and Gemini surfactant-based delivery systems (Mohammed Siddiq et al., 2019; Parikh et al., 2015) demonstrate low toxicity toward non-cancerous cells.

In contrast, in A431 cancer cells, OF12 displayed enhanced anticancer efficacy, with an IC₅₀ of 0.77 ± 0.02 μg/mL, followed by OF16 (1.04 ± 0.11 μg/mL) and pure 5-FU (1.15 ± 0.01 μg/mL). The superior performance of OF12 indicates improved intracellular uptake and sustained release of 5-FU by the cubosomal system, leading to greater inhibition of cancer cell proliferation. The Gemini surfactants showed moderate cytotoxicity in A431 cells, with lower activity than 5-FU, reflecting their limited standalone anticancer potential. Based on the overall cytotoxicity data and improved skin deposition observed in previous studies, OF12 was identified as the lead formulation and was chosen for subsequent studies.

#### 3.8.2 Cell uptake studies

Flow cytometry analysis revealed a significantly higher uptake of OF12 compared to pure 5-FU in A431 cells, as indicated by increased FITC fluorescence intensity **(**Fig. 5C**).** Histograms of individual samples further confirmed enhanced internalization of the cubosomal formulation **(**Fig. 5D-F**).** This improved uptake is likely attributed to the positive surface charge of OF12, which promotes stronger electrostatic interactions with the negatively charged cell membrane, facilitating efficient internalization (Augustine et al., 2020; Lin and Alexander-Katz, 2013). These findings correlate with the cytotoxicity results, where enhanced cellular uptake of OF12 was associated with greater anticancer efficacy compared to free 5-FU.

#### 3.8.3 Apoptosis assay

The AO/EB staining assay demonstrated that OF12 cubosomes induced a significantly greater degree of apoptosis and necrosis in A431 cells compared to free 5-FU. Cells treated with OF12 showed prominent red/orange fluorescence, nuclear fragmentation, and membrane blebbing—hallmarks of late apoptosis and necrosis **(**Fig. 5G**)**. In contrast, cells treated with 5-FU alone exhibited minimal apoptotic changes, while untreated cells retained their normal morphology and showed uniform green fluorescence, characteristic of healthy, viable cells. These observations are in good agreement with previous reports confirming the apoptotic potential of 5-FU in A431 cells (Rana et al., 2019). Moreover, the enhanced apoptotic response observed with cubosome-based delivery aligns with earlier findings on the improved efficacy of nanocarriers, such as icariin-loaded cubosomes in SKOV-3 cells (Fahmy et al., 2020).

#### 3.8.4 OF12-GEL induces G₂/M Phase arrest and enhances anti-proliferative activity

Flow cytometry analysis revealed significant alterations in cell cycle distribution following treatment. OF12-GEL induced a pronounced arrest in the G₂/M phase, with 49.3% of cells halted at this stage, compared to 40.65% for pure 5-FU and 25.35% in the untreated group **(**Fig. 5H**)**. This shift indicates a robust anti-proliferative effect of the cubosomal formulation. A reduction in G₀/G₁ phase population was observed in treated groups, with OF12-GEL showing the lowest percentage (41.26%) compared to 49.8% (5-FU) and 69.04% (control), reflecting inhibited cell cycle progression. The sub-G₀/G₁ phase, representing apoptotic cells, increased to 4.27% and 5.32% for OF12-GEL and 5-FU, respectively, compared to 1.36% in the control **(**Fig. 5I-K**)**. These findings suggest that OF12-GEL effectively induces G₂/M phase arrest, thereby suppressing proliferation of A431 cells more efficiently than free drug. The observed findings are in agreement with earlier reports, which have shown that 5-FU exerts its anticancer effect by obstructing cell cycle progression, particularly at the G₂/M phase (Ombredane et al., 2021; Peng et al., 2022).

### 3.9 *In vivo* safety and efficacy studies

#### 3.9.1 Visual and histopathological assessment confirms dermal safety of OF12-GEL

**Fig. 6:**
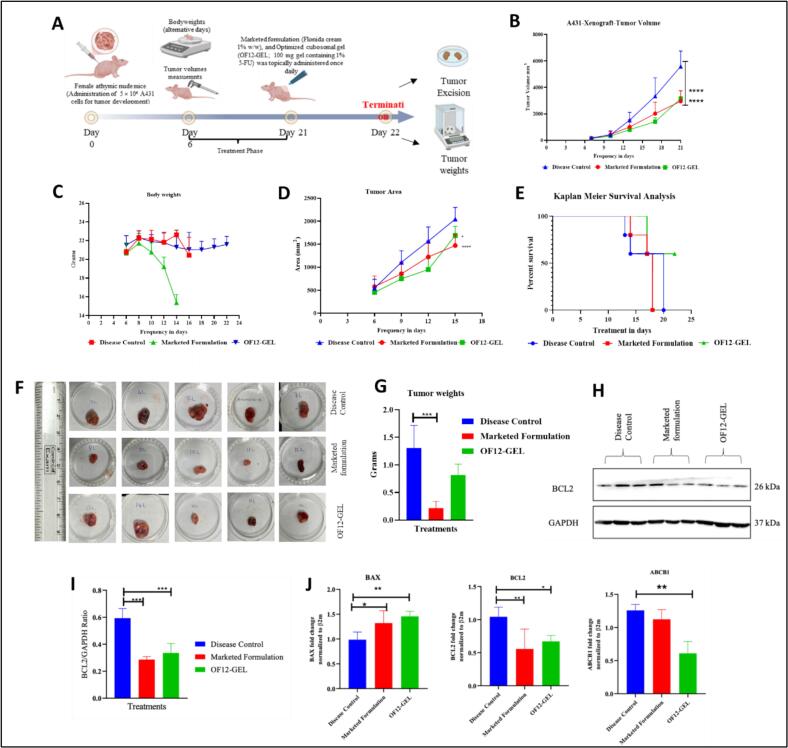
(A) Schematic representation of tumor induction and treatment timeline. (B) Tumor growth curves after various treatments (*n*=6). (C) Changes in body weight with time. (D) Tumor area after various treatments. (E) Survival curves of A431 tumor-bearing mice with different treatments. (F) Photographs of the tumors from various treatment groups. (G) Tumor wights after various treatments. (H) Western blot analysis of BCL2 protein expression post different treatments. (I) Expression of BCL-2 in disease control, marketed formulation and OF12-GEL treated groups. (J) Expression of BAX, BCL2, and ABCB1 for disease control, marketed formulation and OF12-GEL treated groups. Data represent mean ± SD (*n* = 3). *p < 0.05, **p < 0.01 and ***p < 0.001.

The dermal safety profile of OF12-GEL was assessed through visual observation and histopathological examination following topical application in wistar rats. No alterations in skin texture or color were noted in the control group. The placebo formulation and OF12-GEL treated groups exhibited skin appearance comparable to the untreated control, indicating good tolerability. In contrast, the positive control group (0.8% formalin) exhibited severe signs of irritation, including redness, inflammation, and scar formation **(**Fig. 6**).** The Primary Irritation Index (PII) scores further substantiated these findings. The PII values for the formalin-treated, placebo, and OF12-GEL groups were 0.83, 0.16, and 0.33, respectively (Table S14). As the values remained below the irritancy threshold (PII < 0.5), OF12-GEL was classified as non-irritant. These results are supported by previous literature demonstrating the biocompatibility of cubosomal systems for dermal delivery (Boge et al., 2019; Janakiraman et al., 2019b).

Histopathological analysis (Fig. S6) provided additional confirmation. Skin sections from the control group displayed normal architecture with a thin, stratified squamous keratinized epithelium and absence of edema, fibrosis, or inflammatory cell infiltration. In contrast, the formalin-treated group showed marked epidermal damage, including ulcerative epithelium, neutrophilic aggregates, and increased epithelial thickness (3–7 cell layers), along with both acute and chronic inflammation in the dermis. Skin sections from both placebo and OF12-GEL-treated groups closely resembled those of the untreated control, without any pathological alterations. Grading of histological parameters (Table S15) supported the visual findings, confirming the non-toxic and non-irritant nature of the developed formulation. Collectively, the absence of dermal abnormalities in both macroscopic and microscopic evaluations affirms the safety of OF12-GEL for topical administration. These observations align with prior reports that have highlighted the dermal compatibility and low irritancy of cubosome-based formulations (Manikkath et al., 2020; Peng et al., 2015).

#### 3.9.2 FTIR spectroscopic analysis of the stratum corneum

To assess the potential effect of the gemini surfactant on stratum corneum lipid and protein structure, FTIR spectroscopy was employed to compare untreated SC (PBS-treated) and SC exposed to the Gemini surfactant (Fig. S7). The FTIR spectra of PBS-treated SC revealed characteristic absorption peaks at 2919.56 cm^−1^ and 2850.85 cm^−1^, corresponding to CH₂ asymmetric and symmetric stretching vibrations, respectively. Additionally, prominent amide I and amide II bands were noted at 1639.89 cm^−1^ and 1541.56 cm^−1^, representing the proteinaceous keratin matrix. Notably, SC samples treated with the Gemini surfactant showed minimal shifts in characteristic peaks, indicating preserved lipid bilayer structure and protein conformation. Unlike conventional enhancers that disrupt SC integrity (Obata et al., 2010), the Gemini surfactant exhibited mild, non-disruptive interactions, suggesting its potential as a safe and biocompatible transdermal permeation enhancer.

#### 3.9.3 Antitumor efficacy of OF12-GEL in DMBA-induced squamous cell carcinoma rat model

Histopathological evaluation confirmed successful induction of squamous cell carcinoma (SCC) in the positive control group, characterized by hyperkeratosis, ulcerative epithelium, connective tissue invasion by epithelial islands, keratin pearl formation, nuclear pleomorphism, and a high mitotic index (Fig. S9). Treatment in all groups was initiated only after uniform tumor formation was confirmed histologically, despite minor visual differences in macroscopic lesions at Day 0 due to natural variability. In contrast, the normal control group exhibited well-organized epidermal and dermal structures, with no signs of neoplastic changes. Treatment with the Flonida cream 1% *w*/w resulted in partial tumor regression; however, histological sections revealed persistent epithelial ulceration, limited clearance of epithelial islands from connective tissue, and mild inflammatory infiltration. One to two mitotic figures per high-power field were still observed (Table S16), suggesting incomplete therapeutic resolution.

Remarkably, the OF12-GEL-treated group demonstrated a substantial reduction in neoplastic features. Histological sections revealed minimal epithelial ulceration, absence of epithelial islands in connective tissue, and active dermal remodeling characterized by organized collagen bundles with both acute and chronic inflammatory cell infiltration. The absence of keratin pearls and mitotic figures supported complete tumor regression. These findings were consistent with visual assessments, which showed a significant reduction in the SCC-affected area in OF12-GEL-treated animals compared to both the untreated and marketed formulation groups**.**

Though fluorouracil is a widely used first-line chemotherapeutic agent, but its dose-limiting toxicities markedly impair patient quality of life and often require dose reductions that compromise therapeutic efficacy. 5-FU–induced mucositis is well documented (Astuti et al., 2025; Kim et al., 2018), and the pronounced decline observed in the marketed 5-FU group is consistent with established *in vivo* evidence of severe local tissue injury, including ulceration, hemorrhage, edema, and epithelial disruption. Additionally, 5-FU causes substantial body-weight loss (Hamouda et al., 2017), with obese mice exhibiting ∼15% reduction compared to ∼12% in non-obese mice, along with reports of elevated mortality (VanderVeen et al., 2022)

In our study, a similarly marked body-weight reduction was noted with the marketed formulation, whereas OF12L gel and the disease-control group showed comparatively low side effects. The superior antitumor efficacy of OF12-GEL can be attributed to its cubosomal architecture, which facilitates prolonged retention and enhanced accumulation at the tumor site, enabling sustained 5-FU release and efficient therapeutic action. These results align with previous reports highlighting the potential of cubosomal systems in dermal malignancies (Chen et al., 2023).

#### 3.9.4 Antitumor efficacy of OF12-GEL in A431 xenografted nude mice

The antitumor efficacy of OF12-GEL was evaluated in A431 xenograft-bearing nude mice to determine its therapeutic potential **(**Fig. 6A**)**. Visual examination of excised tumors **(**Fig. 6F**)** demonstrated a marked reduction in tumor size in both the OF12-GEL and Flonida cream 1% w/w treated groups compared to the disease control. Quantitative analysis of tumor volumes recorded throughout the study **(**Fig. 6B**)** confirmed significant tumor growth inhibition by both treatments. Notably, the OF12-GEL group exhibited a more pronounced decrease in tumor volume relative to the marketed formulation, highlighting its superior anticancer activity. In addition to tumor volume, quantitative assessment of tumor area **(**Fig. 6D**)** revealed a significant reduction in the OF12-GEL–treated group compared to the disease control. Furthermore, excised tumor weight analysis **(**Fig. 6G**)** demonstrated a statistically significant decrease in tumor mass in the OF12-GEL group, corroborating the volumetric and areal findings and confirming effective tumor growth suppression.

Body weight monitoring during treatment **(**Fig. 6C**)** showed that mice treated with OF12-GEL maintained significantly higher weights than those in the disease control and marketed formulation groups, indicating lower systemic toxicity and improved tolerability. This suggests that OF12-GEL treatment better preserves overall health compared to other groups, consistent with findings reported earlier. (Safwat et al., 2018).

Survival analysis further demonstrated the superior therapeutic efficacy of OF12-GEL **(**Fig. 6E and Table S17). All animals in the untreated control group exhibited the shortest survival times, while those treated with the marketed formulation showed a modest extension in lifespan. In contrast, treatment with OF12-GEL significantly prolonged survival, with 60% of mice remaining alive at the study endpoint (*p* < 0.05 *vs.* control). This enhanced survival is attributed to the formulation's improved skin retention, sustained drug release, and targeted delivery, which collectively contribute to increased antitumor activity. Although both OF12-GEL and the marketed formulation reduced tumor size, the OF12-GEL group consistently demonstrated greater tumor volume suppression, improved survival, and better preservation of body weight parameters derived from quantitative measurements, which underscore its superior efficacy despite only modest visual differences observed in excised tumors **(**Fig. 6F**)**. While histopathology of different organs was not performed, the absence of body weight loss, behavioural changes, or treatment-related deaths suggests a favorable safety profile, to be confirmed in future studies.

#### 3.9.5 Regulation of BAX, BCL-2, and ABCB1 expression by OF12-GEL correlates with enhanced anti-cancer activity

The OF12-GEL formulation exhibited a pronounced ability to modulate critical regulators of apoptosis and tumor progression. A significant upregulation of the pro-apoptotic gene BAX was observed, suggesting enhanced activation of intrinsic apoptotic pathways compared to both the marketed formulation and disease controls **(**Fig. 6J**)**. This shift was complemented by a notable downregulation of BCL-2, an anti-apoptotic protein, indicating a favorable alteration in the apoptotic balance that favours cancer cell death **(**Fig. 6H and J**)** (Manga et al., 2022). Such modulation of the BAX/BCL-2 axis is a hallmark of effective anti-cancer therapy and underscores the potential of OF12-GEL to trigger programmed cell death (Isacescu et al., 2023). BCL-2/GAPDH ratio was plotted for each treatment group **(**Fig. 6I**).** Importantly, the expression of ABCB1, a gene frequently associated with aggressive tumor phenotypes and poor therapeutic outcomes (Skinner et al., 2023), was significantly suppressed following OF12-GEL treatment **(**Fig. 6J**)**. This reduction may enhance the cytotoxic efficacy of the treatment by mitigating tumor cell survival mechanisms (Adorni et al., 2023). Together, these molecular findings not only validate the superior antitumor efficacy of OF12-GEL observed in histopathological and *in vivo* studies but also provide a mechanistic framework explaining its potential to overcome tumor growth and resistance pathways. This multifaceted action positions OF12-GEL as a promising candidate for enhanced cancer therapy.

#### 3.9.6 Histopathological and immunohistochemical evaluation demonstrates antitumor and anti-angiogenic effects of OF12-GEL

**Fig. 7:**
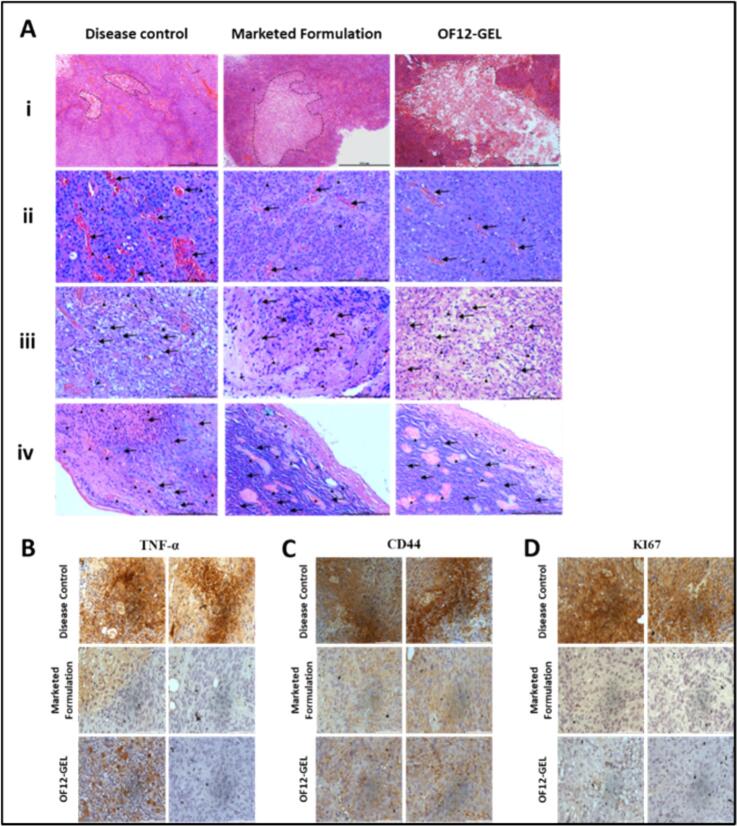
(A) Hematoxylin and Eosin (H&E) staining of the excised tumors for different treatment groups (*n* = 6); (i) necrosis within the tumor stroma (4×); (ii) stromal cellular density, angiogenesis (arrow), and mitotic figures (arrowhead) (20×); (iii) desmoplasia with fibroblasts (arrow) and inflammatory cell infiltration (arrowhead) (20×); (iv) dermal invasion with neoplastic cell infiltration (arrow), inflammatory response (asterisk), and neovascularization (arrowhead) (20×). Immunohistochemistry analysis of (B) TNF-α, (C) CD44 (D) Ki67 in the whole tumor tissues of A431 tumor-bearing nude mice post different treatments.

Histological analysis showed extensive necrosis in the treated groups, marked by amorphous pale eosinophilic areas with peripheral inflammatory infiltration **(**Fig. 7A **i)**. In contrast, the disease control group exhibited dense neoplastic proliferation, pronounced neovascularization, and inflammatory cell margination, indicating active tumor progression **(**Fig. 7A **ii)**. Both the marketed formulation and OF12-GEL significantly reduced stromal density, vascularization, and mitotic activity, with the absence of inflammatory cell margination. Fig. 7A **iii** shows prominent fibroblast proliferation and fibro-inflammatory changes in the disease control group, which were markedly reduced in the treated groups. As seen in Fig. 7A **iv**, dermal invasion, inflammation, and vascularization were extensive in the disease control group but attenuated in treated groups, with OF12-GEL showing superior reduction. Histological scores (Table S18) confirmed improved outcomes in both treatment groups, with OF12-GEL being more effective. Microvessel density (Table S19) was highest in disease control, lower with the marketed formulation, and lowest with OF12-GEL, highlighting its antiangiogenic potential.

Complementing the histological findings, immunohistochemical analysis suggested a notable reduction in the expression of TNF-α **(**Fig. 7B**)**, CD44 **(**Fig. 7C**)**, and Ki67 **(**Fig. 7D**)** following treatment with OF12-GEL and the marketed formulation, compared to their elevated levels in the disease control group. Both treatments exhibited strong anti-cancer activity, with comparable performance across the evaluated markers. The marketed formulation showed slightly greater efficacy in certain instances, the overall findings, supported by *in vivo* studies in mice and rats, affirm the therapeutic potential and safety of OF12-GEL. Due to stringent animal ethics committee restrictions on the number of animals used, a control group was not included. Accordingly, the analysis was focused on evaluating therapeutic effects relative to the disease state, in line with the primary objective of the study.

## 4 Conclusion

In this work, a novel 5-FU-loaded cubosomal gel (OF12-GEL) stabilized with gemini surfactants was successfully developed and optimized for topical treatment of cutaneous malignancies. The optimized cubosomes exhibited a nanoscale size of 134.4 nm, high stability (+76.1 mV), and 51.3% entrapment efficiency. OF12-GEL achieved a markedly sustained release (41% at 24 h) and enhanced skin retention to 323.7 μg/cm^2^, a 2.8-fold increase compared to free 5-FU. *In vitro*, OF12 displayed superior anticancer efficacy against A431 cells, reducing the IC₅₀ to 0.77 μg/mL, inducing G₂/M arrest (49.3% cells), and enhancing cellular uptake compared to free 5-FU. *In vivo*, OF12-GEL significantly inhibited tumor growth in both DMBA-induced SCC rats and A431 xenografted nude mice, reducing tumor burden, improving survival, and downregulating key oncogenic markers (BCL-2, Ki67, TNF-α, ABCB1). Dermal safety studies confirmed its non-irritant nature (PII = 0.33).

Overall, the formulation provides a potent, localized, and patient-friendly alternative to conventional 5-FU creams, overcoming limitations of poor skin retention. OF12-GEL represents a promising candidate for further preclinical development and potential clinical translation.

## CRediT authorship contribution statement

**Ruchira Raychaudhuri:** Writing – review & editing, Writing – original draft, Methodology, Investigation, Formal analysis, Data curation, Conceptualization. **Ajinkya Nitin Nikam:** Methodology, Investigation. **Naitik Jain:** Writing – review & editing, Writing – original draft, Formal analysis, Data curation. **Abhisheik Eedara:** Data curation, Investigation. **Neha Kandpal:** Methodology, Investigation. **Rajdeep Ray:** Methodology, Data curation. **Krishnadas Nandakumar:** Writing – review & editing, Methodology, Formal analysis, Data curation. **Sai Balaji Andugulapati:** Writing – review & editing, Methodology, Formal analysis, Data curation. **Srinivas Mutalik:** Writing – review & editing, Writing – original draft, Validation, Supervision, Resources, Project administration, Formal analysis, Data curation, Conceptualization.

## Ethics approval

This study involving experimental mice was conducted in accordance with adhering to the institutional and national guidelines, and the experimentation protocol was approved by the Institutional Animal Ethical Committee, KMC, Manipal (IAEC/KMC/112/2020) and (IAEC/KMC/94/2022). The experimental protocol for the experiments in nude mice was approved by the Institutional Animal Ethics Committee (IICT/IAEC/055/2022) at CSIR-Indian Institute of Chemical Technology (CSIR-IICT), Government of India, Hyderabad, India.

## Declaration of competing interest

The authors declare no competing interests.

## Data Availability

Data is presented in the main manuscript and the Supplementary Information.

## Appendix A Supplementary data

Supplementary material Image 1
