# Supplementary material for: Gemini surfactant-stabilized cubosomes for enhanced topical delivery of 5-fluorouracil in cutaneous squamous cell carcinoma
Source: Int J Pharm X. 2026 Feb 6;11:100504. doi: 10.1016/j.ijpx.2026.100504 (PMC12908058; doi:10.1016/j.ijpx.2026.100504)
Supplement: Supplementary file 1 — Supplementary material [file mmc1.docx]

**Gemini Surfactant-Stabilised Cubosomes for Enhanced Topical Delivery of 5-Fluorouracil in Cutaneous Squamous Cell Carcinoma**

Ruchira Raychaudhuri^1^, Ajinkya Nitin Nikam^1^, Naitik Jain^1^, Abhisheik Eedara^2^, Neha Kandpal^1^, Rajdeep Ray ^3^, Krishnadas Nandakumar ^4^, Sai Balaji Andugulapati^2^, Srinivas Mutalik^1*^

^1^ Department of Pharmaceutics, Manipal College of Pharmaceutical Sciences, Manipal Academy of Higher Education, Manipal 576104, Karnataka, India

^2^Department of Applied Biology, CSIR-Indian Institute of Chemical Technology (CSIR-IICT), Hyderabad, 500007, Telangana, India

^3^ Department of Pharmaceutical Chemistry, Manipal College of Pharmaceutical Sciences, Manipal Academy of Higher Education, Manipal 576104, Karnataka, India

^4^Department of Pharmacology, Manipal College of Pharmaceutical Sciences, Manipal Academy of Higher Education, Manipal 576104, Karnataka, India

***Corresponding Author**

Dr Srinivas Mutalik

Professor and Principal

Manipal College of Pharmaceutical Sciences

Manipal Academy of Higher Education

Manipal 576104, Karnataka, India

Email: [ss.mutalik@manipal.edu](mailto:ss.mutalik@manipal.edu)

1. **Methodology**
   1. **Synthesis of gemini surfactants**

Gemini surfactants, GS12 and GS16, were synthesized via a two-step procedure involving alkylation and subsequent quaternization, based on previously reported methods with minor modifications Thin-layer chromatography was used to monitor the reaction progress and assess the purity of the final compounds.

**Step 1: Synthesis of Alkylated Intermediate**

A mixture of 2-(methylamino) ethanol and either 1-bromododecane (for GS12) or 1-bromohexadecane (for GS16) was stirred in a 3-necked round-bottom flask at 90 °C for 12–14 hours. Upon completion, excess 2-(methylamino)ethanol was removed under reduced pressure. The upper phase of the resulting biphasic mixture was separated, dissolved in dichloromethane (DCM), and washed sequentially with aqueous NaOH and brine. The organic layer was dried over anhydrous sodium sulfate, filtered, and concentrated to yield the crude intermediate.

**Step 2: Quaternization Reaction**

The intermediate was dissolved in anhydrous acetone, and 1,4-dibromobutane was added dropwise. The reaction mixture was stirred at 60 °C for 70 hours, then cooled in an ice bath and refrigerated overnight. The solvent was removed, and the residue was washed with a 1:1 mixture of petroleum ether and ethyl acetate. The crude product was filtered using a Buchner funnel and recrystallized from methanol to obtain the final Gemini surfactants.

**S1.2 HPLC method development and validation (Analytical and bioanalytical)**

An HPLC method for the quantitative estimation of 5-Fluorouracil (5-FU) was developed using a Shimadzu HPLC system with UV detection, employing a Phenomenex Luna C18 column (250 × 4.6 mm, 5 μm). The method was optimized by first identifying 265 nm as the detection wavelength through UV scanning, followed by evaluating multiple mobile phase compositions consisting of acetonitrile and acidified water (pH 3.0, adjusted with TFA). A composition of 98:2 (acidified water:acetonitrile, v/v) at a flow rate of 0.8 mL/min produced the most acceptable retention time, peak symmetry, and theoretical plates. Standard solutions were prepared in phosphate buffer (pH 7.4), and the method was validated according to ICH Q2(R1). For bioanalysis, the same chromatographic conditions were used, and liquid–liquid extraction with methyl tert-butyl ether (MTBE) was optimized after protein precipitation failed to adequately retain 5-FU. Rat plasma was spiked with 5-FU and the internal standard 5-bromouracil, extracted, evaporated at 65°C, and reconstituted in phosphate buffer prior to injection.

**S2. Results and Discussion**

**S2.1. Drug- Excipient compatibility studies**

The FTIR spectrum of the physical mixture containing TM, Span 60, and Tween 80 (**Fig. S3 C)** exhibited the characteristic peaks of the drug at 3491 cm⁻¹ (O-H stretching of carboxylic acid), 2919 cm⁻¹ (C-H stretching of methyl groups), 1734 cm⁻¹ (C=O stretching of ketone), 1640 cm⁻¹ (C=C stretching in the aromatic ring), 1457 cm⁻¹ (aliphatic C-H bending), 1293 cm⁻¹ (aliphatic C-N stretching), 944 cm⁻¹ (N-H wagging), and 850 cm⁻¹ (C-H bending). Comparison with the spectra of Span 60 (**Fig. S3 A**) and Tween 80 (**Fig. S3 B)** confirmed that these peaks remained unaffected in the mixture, indicating no significant chemical interaction or incompatibility between the drug and the excipients.

To further confirm this compatibility, DSC analysis was conducted **(Fig. S3 D)**. Pure TM exhibited a sharp endothermic peak at 206°C, corresponding to its melting point and indicating its crystalline nature, while Span 60 showed a peak at 54°C. In the physical mixture, the TM peak shifted slightly to 196°C, and Span 60 melted at 55°C. The reduced intensity of TM’s endothermic peak suggests partial solubilization in molten Span 60 before reaching its melting point. The minor decrease in TM’s melting temperature is likely due to the moistening effect of the excipient, further supporting the absence of any significant interaction and confirming the chemical stability of the drug in the presence of Span 60.

**S2.2. HPLC Analytical Method Development and Validation**

The optimized mobile phase (98:2, acidified water:acetonitrile) produced a well-resolved peak for 5-FU with satisfactory theoretical plates and minimal tailing. The 0.8 mL/min flow rate further enhanced resolution, preventing overlap with the diluent peak and ensuring consistent retention **(Table S20)**. The calibration curve demonstrated excellent linearity over 1–10 µg/mL with R² = 0.9987, confirming proportionality between concentration and peak area. The LOD (72 ng/mL) and LOQ (100 ng/mL) indicated high method sensitivity suitable for routine analyses.

Accuracy across three levels (1, 3, and 5 µg/mL) ranged from 89.8% to 99.3%, reflecting reliable recovery. Intra-day and inter-day precision values (%RSD < 2%) complied with ICH limits, demonstrating method robustness. These results confirm that the analytical method is precise, accurate, and fit for quantitative analysis of 5-FU **(Table S21)**.

**S2.3. Bioanalytical Method Development and Validation**

A bioanalytical RP-HPLC method was developed and partially validated for quantification of 5-FU in rat plasma following ICH M10 guidelines. To ensure consistency, the chromatographic conditions established for the analytical method (mobile phase, flow rate, wavelength, and column) were retained for plasma analysis.

Rat plasma samples were collected in EDTA tubes, centrifuged, and stored at −80°C until analysis. Initial attempts using protein precipitation with chilled acetonitrile or methanol resulted in poor analyte retention. Therefore, liquid–liquid extraction (LLE) using methyl tert-butyl ether (MTBE) was optimized. Plasma samples (95 µL) were spiked with 5-FU and the internal standard 5-bromouracil (5-BrU), selected for its structural similarity and suitable elution profile. Following phase separation, the organic layer was evaporated at 65°C, and the residue was reconstituted in phosphate buffer (pH 7.4) before injection. Under the optimized conditions, 5-FU (2500 ng/mL) eluted at 4.3 ± 0.4 min with theoretical plates of 6721 and a tailing factor of 1.72. The ISTD (5-BrU) eluted at 7.37 min with 6586 theoretical plates. No endogenous interference was observed in blank plasma samples **(Figure S8)**.

System suitability (n = 6) met acceptance criteria with %RSD of 1.14%, theoretical plates > 2000, and tailing factor < 1.5. Linearity was observed over 150–10,000 ng/mL (R² = 0.9971). Accuracy and precision were assessed at LQC (250 ng/mL), MQC (1 µg/mL), and HQC (10 µg/mL). Accuracy values ranged from 98.3% to 107.9%, while intra- and inter-batch precision (%CV) ranged from 3.8% to 6.6%, all within ICH M10–specified limits **(Table S22)**. The method was therefore deemed suitable for preclinical pharmacokinetic studies.

**Figures**

**
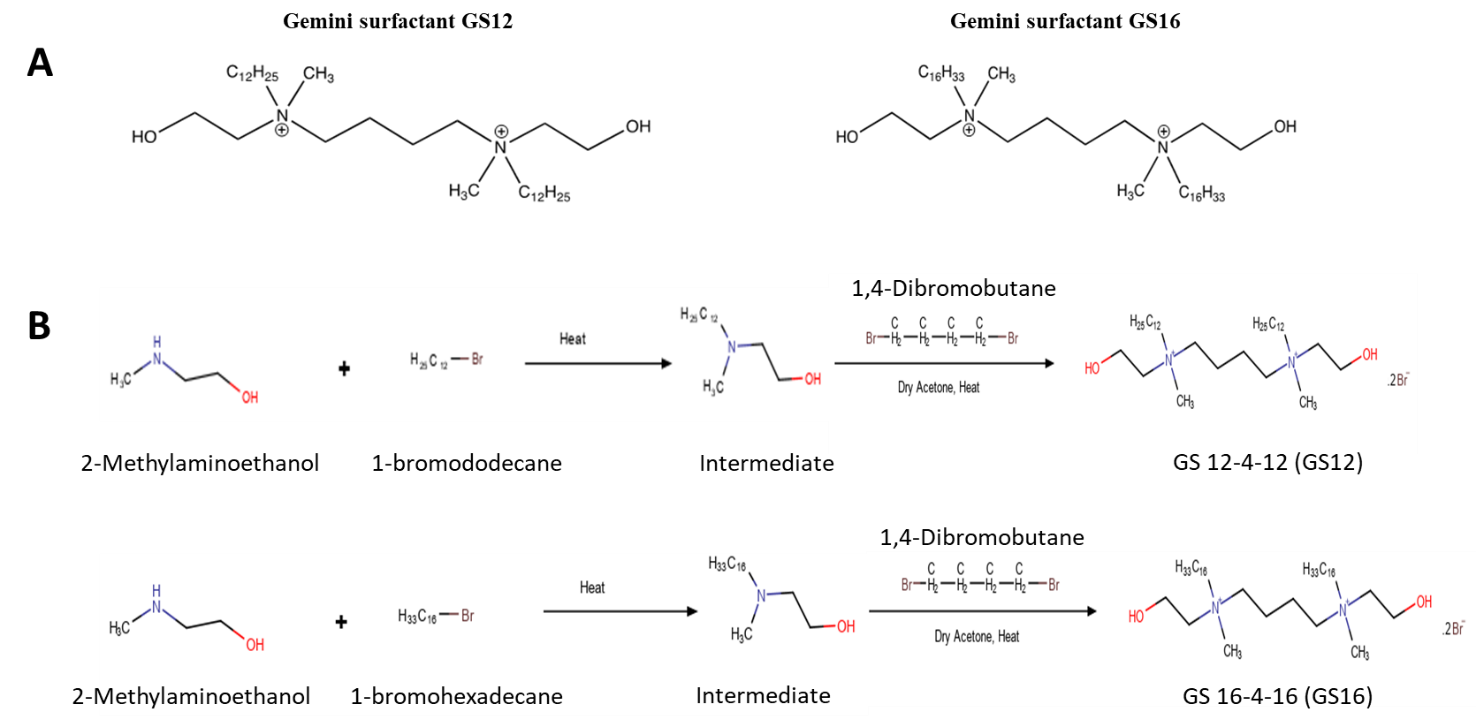
**

**Fig. S1.** **(A) Chemical structures of gemini surfactants GS12 and GS16. (B) Synthetic scheme illustrating the stepwise synthesis of GS12 and GS16 via alkylation and quaternization reactions starting from appropriate diamine and alkyl halide precursors.**

**
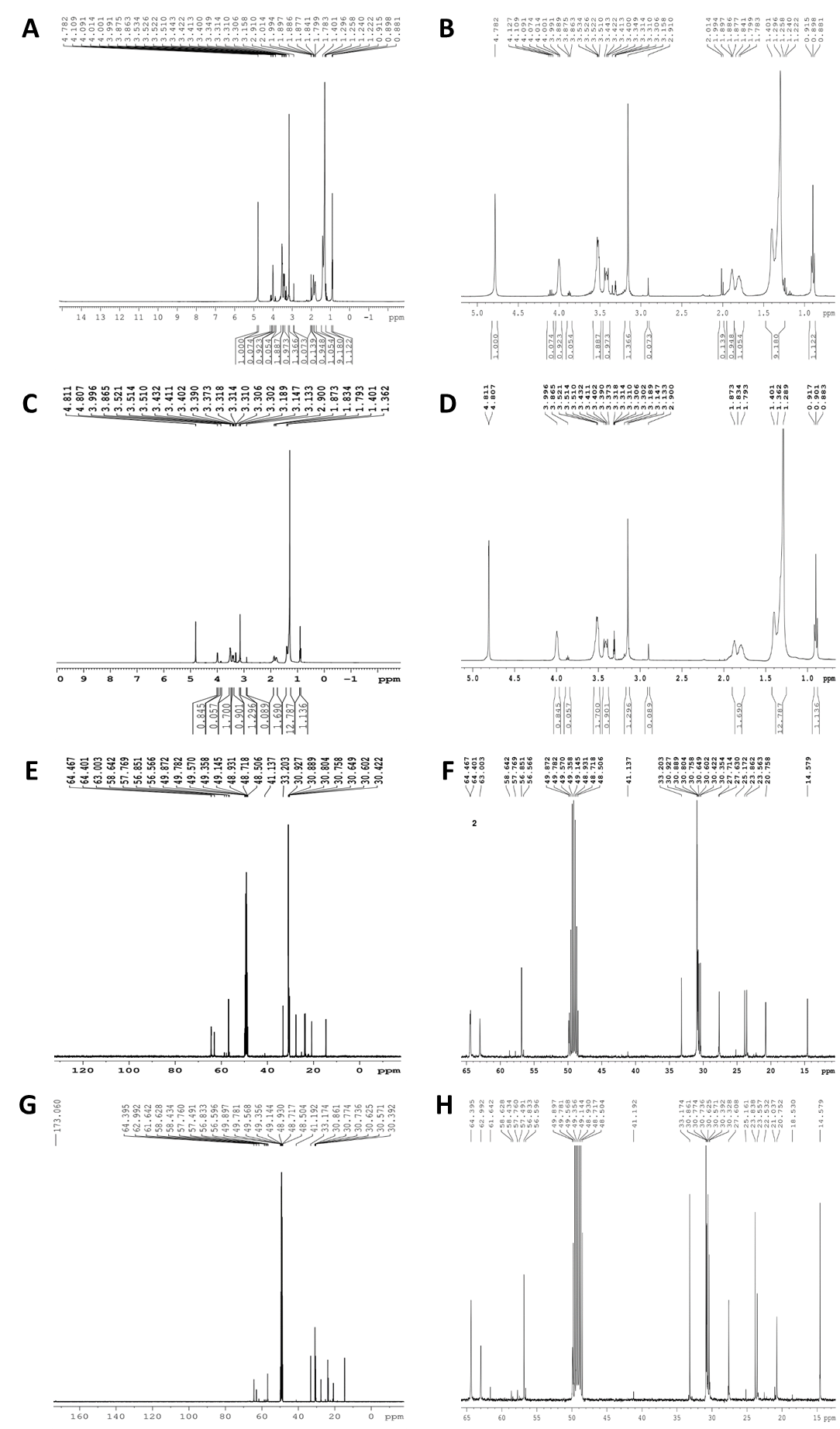
**

**Fig. S2.** **¹H and ¹³C NMR spectra of gemini surfactants GS12 and GS16. (A) ¹H NMR spectrum of GS12; (B) expanded ¹H region of GS12; (C) ¹H NMR spectrum of GS16; (D) expanded ¹H region of GS16; (E) ¹³C NMR spectrum of GS12; (F) expanded ¹³C region of GS12; (G) ¹³C NMR spectrum of GS16; (H) expanded ¹³C region of GS16.**


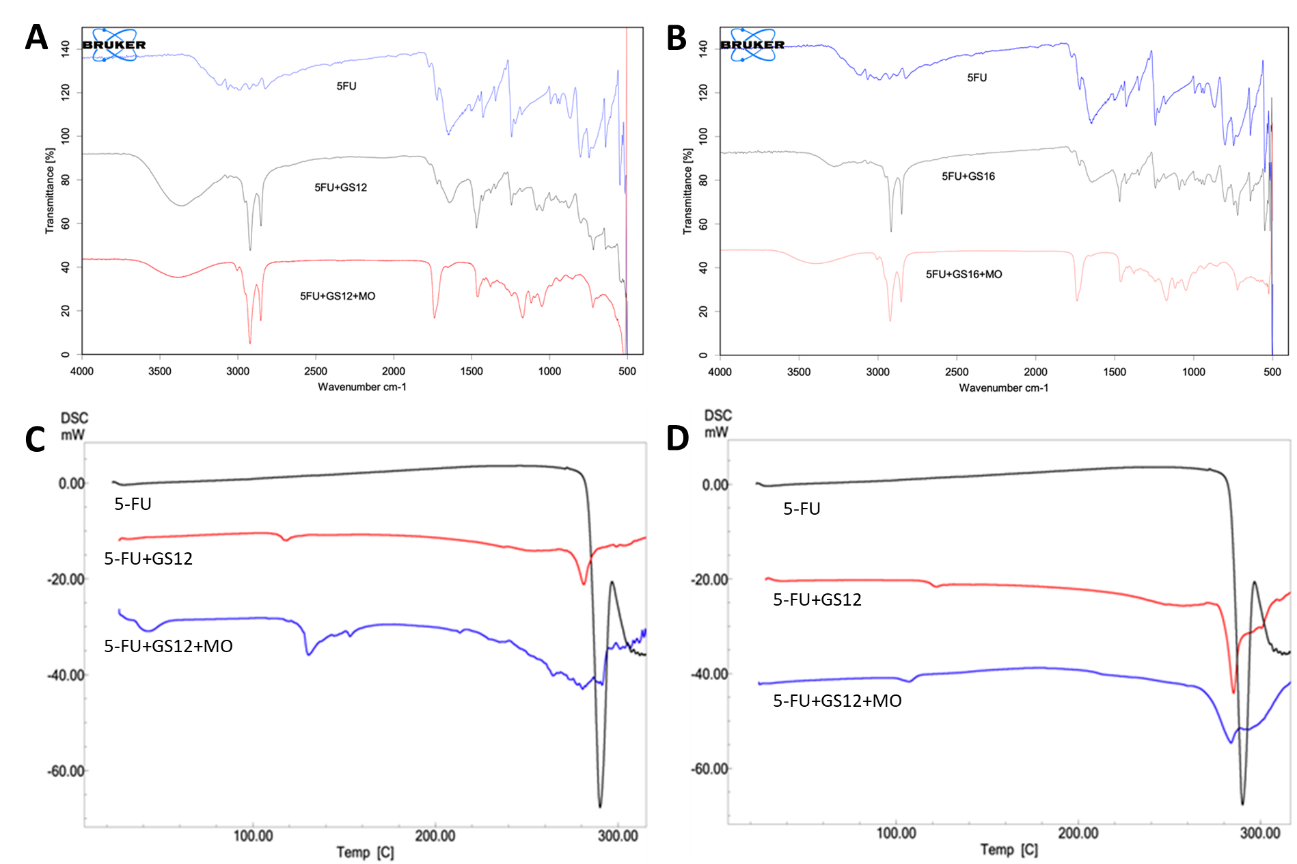


**Fig. S3.** **Characterization of 5-FU and its physical mixtures with GS12 or GS16 and monoolein. (A, B) FTIR spectra of pure 5-FU, 5-FU + GS12 or GS16, and 5-FU + GS12 or GS16 + MO. (C, D) DSC thermograms of pure 5-FU, 5-FU + GS12 or GS16, and 5-FU + GS12 or GS16 + MO.777**


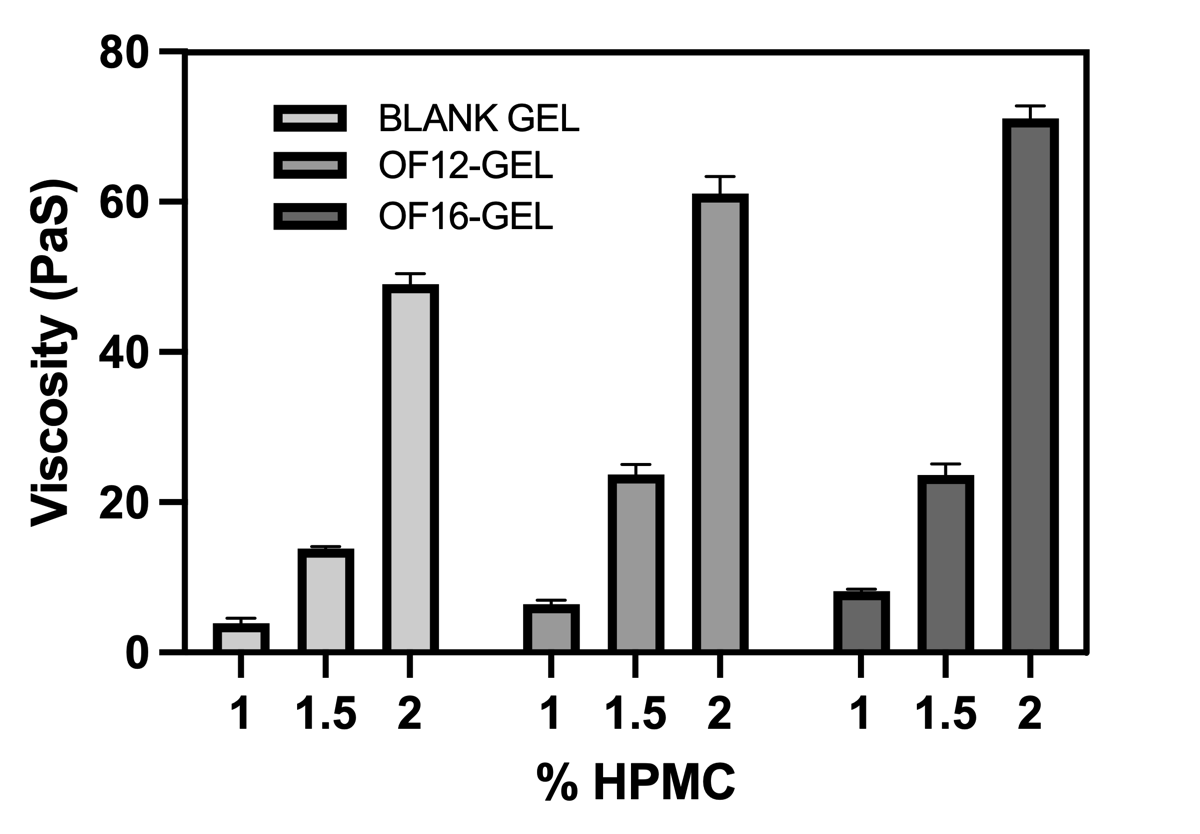


**Fig. S4.** **Viscosity of blank, OF12 and OF16 gels at different HPMC concentrations (% w/w).**

**
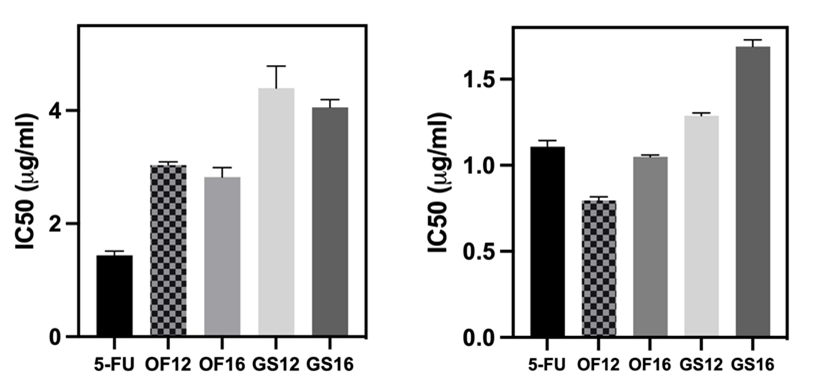
**

**Fig. S5.** **IC₅₀ values of pure drug, optimized formulations (OF12, OF16), and synthesized dimeric surfactants (GS12, GS16) in (A) HaCat and (B) A431 cell lines.**


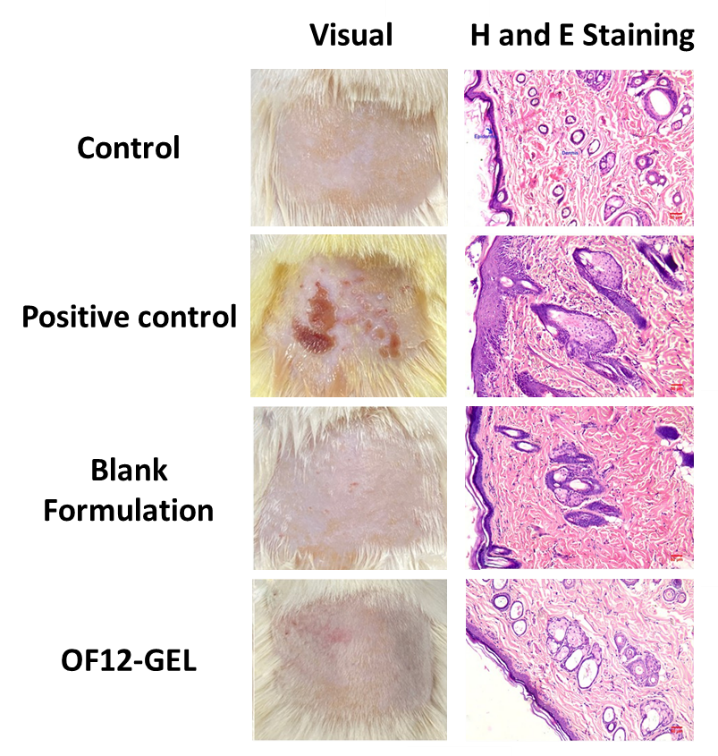


**Fig. S6. Macroscopic and H&E-stained images of rat skin from the skin irritation study, representing the untreated control, positive control (0.8% formalin), blank formulation, and OF12-GEL-treated groups.**

**
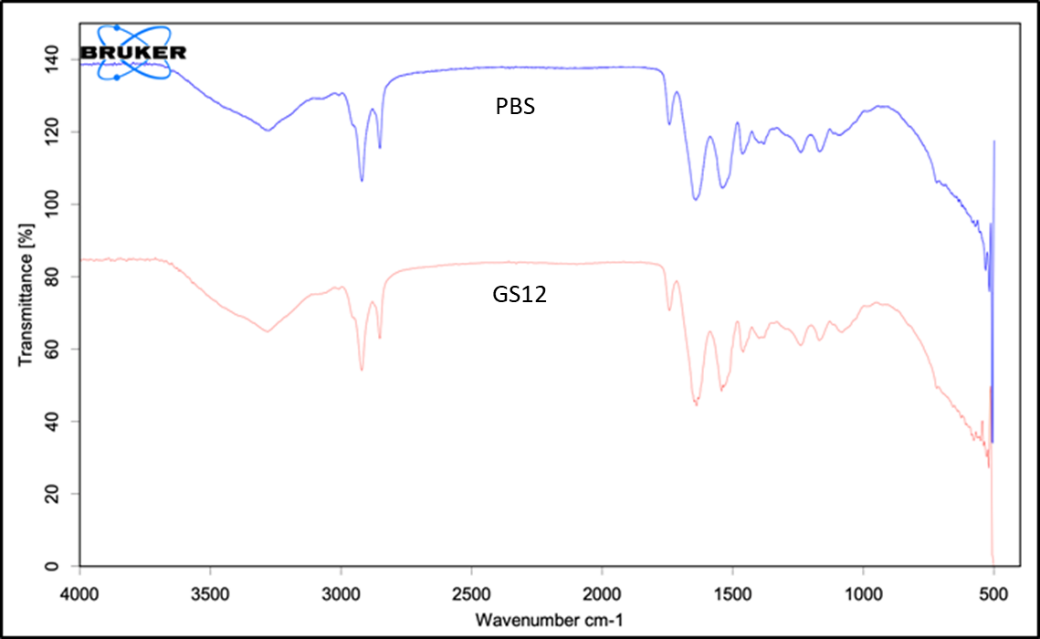
**

**Fig. S7. FTIR spectra of stratum corneum treated with PBS and GS12.**


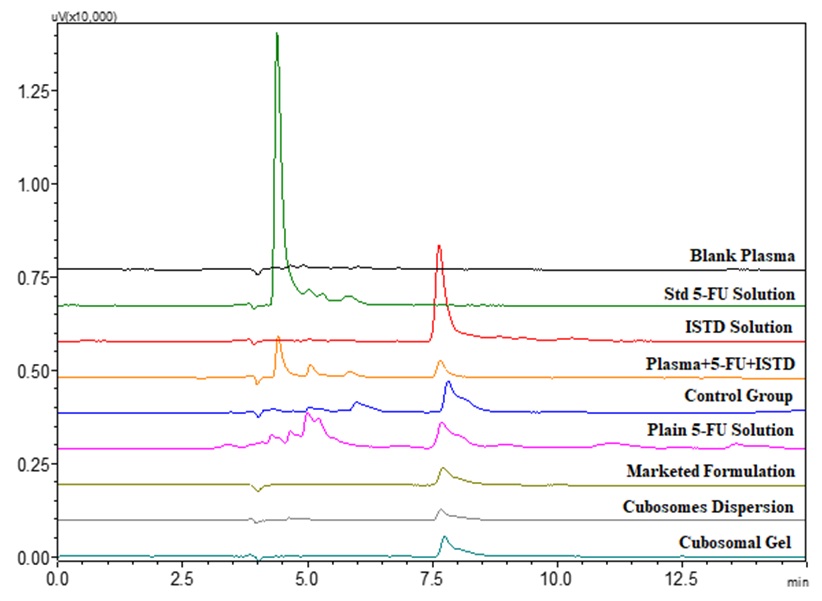


**Fig. S8.** **Overlaid chromatograms of different samples in pharmacokinetics study**


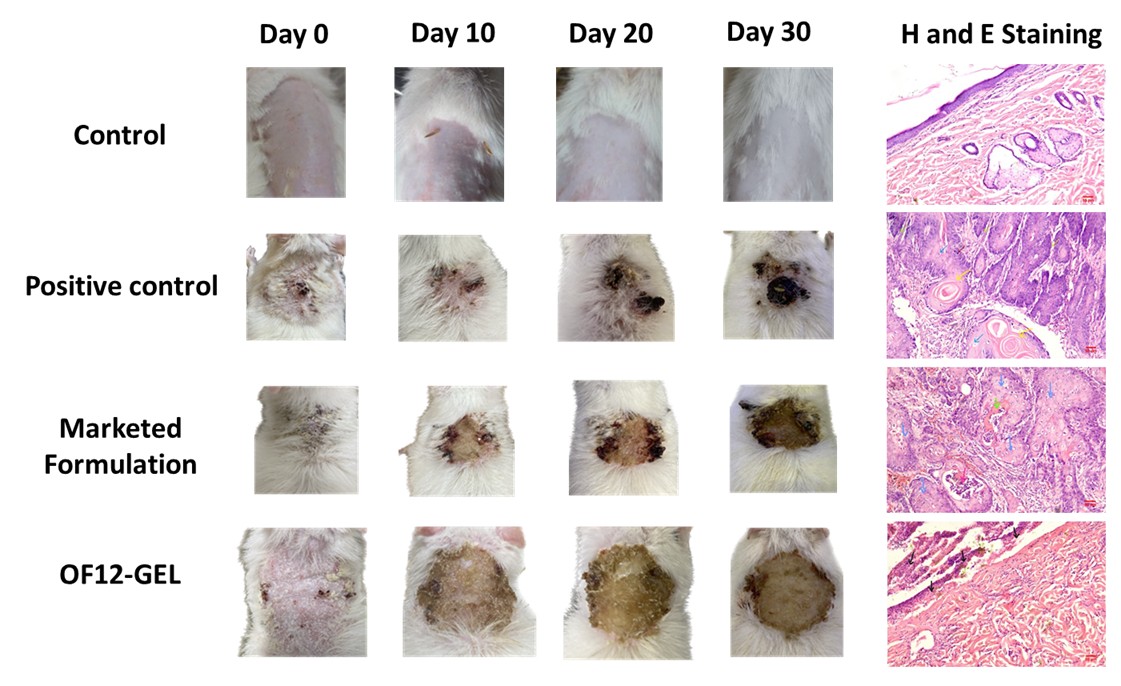


**Fig. S9.** **Macroscopic evaluation and H&E-stained images of rat skin from different treatment groups in the pharmacodynamic study. (Black arrow: Ulcerative epithelium; Pink arrow: Abscess; Green: Keratinisation; Blue arrow: Epithelial Island)**

**
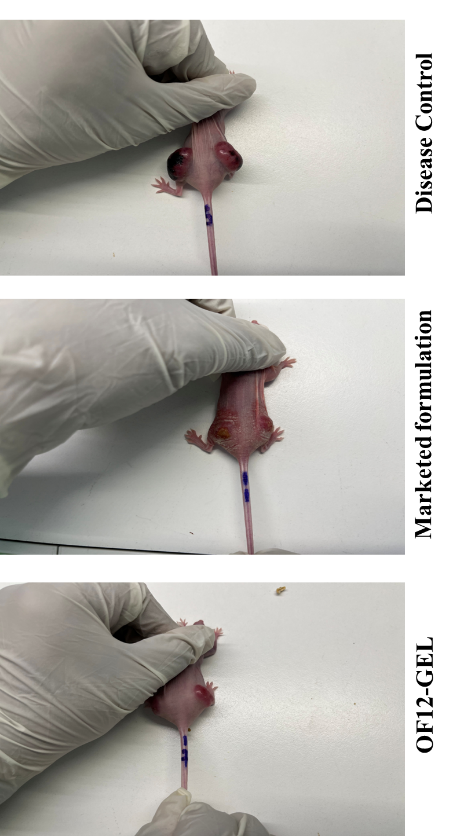
**

**Fig. S10. Representative in situ tumor images on Day 12.**

**Tables**

**Table S1. Composition of cubosomal formulations for optimization by OFAT approach**

| **Formulation code** | **Surfactant** | **Ratio of lipid: surfactant** | **Amount of drug (mg)** | **Homogenization speed (rpm)** | **Homogenization time (minutes)** |
| --- | --- | --- | --- | --- | --- |
| F1 | GS12 | 9.5: 0.5 | 20 | 10000 | 5 |
| F2 |  | 9:1 | 20 | 10000 | 5 |
| F3 |  | 8:2 | 20 | 10000 | 5 |
| F4 | GS16 | 9.5: 0.5 | 20 | 10000 | 5 |
| F5 |  | 9:1 | 20 | 10000 | 5 |
| F6 |  | 8:2 | 20 | 10000 | 5 |
| F7 | GS12 | 9:1 | 10 | 10000 | 5 |
| F8 |  | 9:1 | 20 | 10000 | 5 |
| F9 |  | 9:1 | 30 | 10000 | 5 |
| F10 |  | 9:1 | 40 | 10000 | 5 |
| F11 |  | 9:1 | 50 | 10000 | 5 |
| F12 |  | 9:1 | 75 | 10000 | 5 |
| F13 | GS16 | 9:1 | 10 | 10000 | 5 |
| F14 |  | 9:1 | 20 | 10000 | 5 |
| F15 |  | 9:1 | 30 | 10000 | 5 |
| F16 |  | 9:1 | 40 | 10000 | 5 |
| F17 |  | 9:1 | 50 | 10000 | 5 |
| F18 |  | 9:1 | 75 | 10000 | 5 |
| F19 | GS12 | 9:1 | 30 | 8000 | 2.5 |
| F20 |  | 9:1 | 30 | 8000 | 5 |
| F21 |  | 9:1 | 30 | 8000 | 7.5 |
| F22 |  | 9:1 | 30 | 10000 | 2.5 |
| F23 |  | 9:1 | 30 | 10000 | 5 |
| F24 |  | 9:1 | 30 | 10000 | 7.5 |
| F25 |  | 9:1 | 30 | 12000 | 2.5 |
| F26 (OF12) |  | 9:1 | 30 | 12000 | 5 |
| F27 |  | 9:1 | 30 | 12000 | 7.5 |
| F28 | GS16 | 9:1 | 30 | 8000 | 2.5 |
| F29 |  | 9:1 | 30 | 8000 | 5 |
| F30 |  | 9:1 | 30 | 8000 | 7.5 |
| F31 |  | 9:1 | 30 | 10000 | 2.5 |
| F32 |  | 9:1 | 30 | 10000 | 5 |
| F33 |  | 9:1 | 30 | 10000 | 7.5 |
| F34 |  | 9:1 | 30 | 12000 | 2.5 |
| F35 |  | 9:1 | 30 | 12000 | 5 |
| F36 (OF16) |  | 9:1 | 30 | 12000 | 7.5 |

**Table S2. Composition of different cubosomal gel batches**

| **Gel formulation code** | **Amount of HPMC added** | **Volume of Propylene glycol added** | **Amount of methylparaben added** | **Amount of propylparaben added** |
| --- | --- | --- | --- | --- |
| Blank G1 | 100 mg | 200 μL | 18 mg | 2 mg |
| Blank G2 | 150 mg | 200 μL | 18 mg | 2 mg |
| Blank G3 | 200 mg | 200 μL | 18 mg | 2 mg |
| OF12_G1 | 100 mg | 200 μL | 18 mg | 2 mg |
| OF12_G2 (OF12-GEL) | 150 mg | 200 μL | 18 mg | 2 mg |
| OF12_G3 | 200 mg | 200 μL | 18 mg | 2 mg |
| OF16_G1 | 100 mg | 200 μL | 18 mg | 2 mg |
| OF16_G2 (OF16-GEL) | 150 mg | 200 μL | 18 mg | 2 mg |
| OF16_G3 | 200 mg | 200 μL | 18 mg | 2 mg |

**Table S3. Primary irritation index response classification for irritation**

| **Sl. No.** | **PII** | **Category** |
| --- | --- | --- |
| 1 | < 0.5 | Non-Irritating |
| 2 | 0.5-2 | Slightly Irritating |
| 3 | 2.1-5 | Moderately Irritating |
| 4 | > 5 | Severely Irritating |

**Table S4. δ-value of respective types of H atoms for GS12 surfactant**

| **Type of H** | **Integral** | **Splitting** | **δ-value** |
| --- | --- | --- | --- |
| 6H, -CH_3_ of dodecyl | 1.122 | triplet | 0.881-0.915 |
| 40H, -CH_2_- of dodecyl | 9.180 | multiplet | 1.222-1.401 |
| 6H, N^1^,N^2^-CH_3_ | 1.366 | singlet | 3.158 |
| 4H, 1’,1’’-CH_2_  4H, 2’,2’’-CH_2_  8H, N^1^,N^2^-CH_2_ | 1.887+0.973 | multiplet | 3.306-3.534 |
| 2H, -OH | 0.923 | singlet | 4.001 |
| 4H, 2,3-CH_2_ | 0.748 | multiplet | 1.877-1.897 |


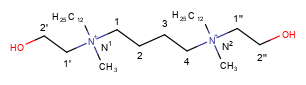


**GS12 surfactant with numbered carbon atoms**

**Table S5. Chemical shift values of respective types of C for GS12 surfactant**

| **Type of C** | **Chemical shift** |
| --- | --- |
| Terminal C of dodecyl | 14.579 |
| CH_3_- N^1^ ,N^2^ – C | 56.833 |
| CH_2_- N^1^ ,N^2^ – C | 61.642 |
| 2’,2’’ OH-C | 64.395 |
| 1’,1’’- C | 62.992 |
| 2,3 –C | 23.838 |
| Dodecyl C | 30.328-30.861 |

**Table S6. δ-value of respective types of H atoms for GS16 surfactant**

| **Type of H** | **Integral** | **Splitting** | **δ-value** |
| --- | --- | --- | --- |
| 6H, -CH_3_ of hexadecyl | 1.136 | triplet | 0.883-0.917 |
| 56H, -CH_2_- of dodecyl | 12.787 | multiplet | 1.289-1.401 |
| 6H, N^1^,N^2^-CH_3_ | 1.296 | singlet | 3.189 |
| 8H, N^1^,N^2^-CH_2_  4H, 1’,1’’-CH_2_  4H, 2’,2’’-CH_2_ | 1.700+0.901 | multiplet | 3.373-3.521 |
| 2H, -OH | 0.845 | singlet | 3.996 |
| 4H, 2,3-CH_2_ | 1.690 | multiplet | 1.793-1.873 |


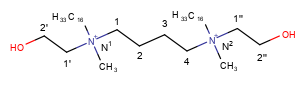


**GS16 surfactant with numbered carbon atoms.**

**Table S7. Chemical shift values of respective types of C for GS16 surfactant**

| **Type of C** | **Chemical shift** |
| --- | --- |
| Terminal C of hexadecyl | 14.579 |
| CH_3_- N^1^ ,N^2^ – C | 56.851 |
| CH_2_- N^1^ ,N^2^ – C | 63.003 |
| 2’,2’’ OH-C | 64.467 |
| 1’,1’’- C | 64.401 |
| 2,3 –C | 23.563 |
| hexadecyl C | 30.354-30.927 |

**Table S8. Particle size, PDI, zeta potential and % entrapment efficiency for variation in HSH speed and time for GS12**

| **Formulation code** | **HSH speed (rpm)** | **HSH time (minutes)** | **Particle size (nm)** | **PDI** | **Zeta potential (mV)** | **% EE of 5-FU** |
| --- | --- | --- | --- | --- | --- | --- |
| F19 | 8000 | 2.5 | 202.5$\pm$1.00 | 0.629$\pm$0.01 | +91.5$\pm$0.7 | 61.4$\pm$2.82 |
| F20 |  | 5 | 176.7$\pm$1.09 | 0.400$\pm$0.03 | +97.3$\pm$0.35 | 62.5$\pm$0.56 |
| F21 |  | 7.5 | 170.4$\pm$0.94 | 0.455$\pm$0.08 | +89.8$\pm$0.87 | 61.1$\pm$2.68 |
| F22 | 10000 | 2.5 | 173.0$\pm$0.5 | 0.409$\pm$0.07 | +95.3$\pm$0.64 | 71.5$\pm$0.77 |
| F23 |  | 5 | 137.7$\pm$0.4 | 0.280$\pm$0.05 | +82.2$\pm$0.79 | 64.6$\pm$1.13 |
| F24 |  | 7.5 | 140.4$\pm$0.6 | 0.256$\pm$0.05 | +76.7$\pm$0.35 | 67.3$\pm$1.48 |
| F25 | 12000 | 2.5 | 187.3$\pm$0.47 | 0.428$\pm$0.03 | +88.3$\pm$1.02 | 65.6$\pm$1.13 |
| F26 (OF12) |  | 5 | 134.4$\pm$1.05 | 0.257$\pm$0.05 | +69.3$\pm$1.27 | 65.7$\pm$1.2 |
| F27 |  | 7.5 | 141.2$\pm$1.55 | 0.269$\pm$0.03 | +75.5$\pm$0.6 | 67.4$\pm$0.49 |

**Table S9. Particle size, PDI, zeta potential and % entrapment efficiency for variation in HSH speed and time for GS16**

| **Formulation code** | **HSH speed (rpm)** | **HSH time (minutes)** | **Particle size (nm)** | **PDI** | **Zeta potential (mV)** | **% Entrapment efficiency of 5FU** |
| --- | --- | --- | --- | --- | --- | --- |
| F28 | 8000 | 2.5 | 181.9$\pm$1.6 | 0.327$\pm$0.01 | +86.5$\pm$1.01 | 50.9$\pm0.14$ |
| F29 |  | 5 | 148.4$\pm$0.9 | 0.192$\pm$0.01 | +67$\pm$1.10 | 50.3$\pm$0.42 |
| F30 |  | 7.5 | 147.3$\pm$1.6 | 0.202$\pm$0.08 | +61.1$\pm$1.11 | 55.3$\pm$0.49 |
| F31 | 10000 | 2.5 | 174.9$\pm$1.7 | 0.366$\pm$0.04 | +81.1$\pm$0.92 | 46.9$\pm$1.9 |
| F32 |  | 5 | 145.1$\pm$0.8 | 0.175$\pm$0.01 | +60.8$\pm$0.75 | 50.8$\pm$2.82 |
| F33 |  | 7.5 | 149$\pm$1.15 | 0.193$\pm$0.01 | +67.8$\pm$0.45 | 44.7$\pm$1.69 |
| F34 | 12000 | 2.5 | 161.1$\pm$2.4 | 0.274$\pm$0.03 | +84.2$\pm$0.98 | 50.1$\pm$0.84 |
| F35 |  | 5 | 137.5$\pm$0.9 | 0.164$\pm$0.05 | +68.3$\pm$1.01 | 43.5$\pm$1.27 |
| F36 (OF16) |  | 7.5 | 141.3$\pm$1.4 | 0.183$\pm$0.06 | +57$\pm$0.75 | 54.5$\pm$0.49 |

**Table S10. Particle size and PDI values of optimized cubosomes OF12 and OF16 before and after lyophilization using different cryoprotectants**

| **Formulation** | **Before lyophilization** | | **Cryoprotectant** | **After lyophilization** | |
| --- | --- | --- | --- | --- | --- |
|  | **Particle size** | **PDI** |  | **Particle size** | **PDI** |
| OF12 | 134.8 | 0.260 | Mannitol (2.5%) | 206.4 | 0.53 |
|  |  |  | Mannitol (5%) | 136.7 | 0.298 |
|  |  |  | Mannitol (10%) | 202.3 | 0.588 |
|  |  |  | Trehalose (2.5%) | 159.5 | 0.42 |
|  |  |  | Trehalose (5%) | 225.8 | 0.672 |
|  |  |  | Trehalose (10%) | 204.7 | 0.592 |
|  |  |  | Sucrose (2.5%) | 174.6 | 0.744 |
|  |  |  | Sucrose (5%) | 183.8 | 0.632 |
|  |  |  | Sucrose (10%) | 194.8 | 0.474 |
| OF16 | 141.8 | 0.220 | Mannitol (2.5%) | 216.3 | 0.441 |
|  |  |  | Mannitol (5%) | 145.3 | 0.316 |
|  |  |  | Mannitol (10%) | 188.3 | 0.54 |
|  |  |  | Trehalose (2.5%) | 152.5 | 0.653 |
|  |  |  | Trehalose (5%) | 196.3 | 0.566 |
|  |  |  | Trehalose (10%) | 204.5 | 0.487 |
|  |  |  | Sucrose (2.5%) | 233.4 | 0.52 |
|  |  |  | Sucrose (5%) | 256.8 | 0.693 |
|  |  |  | Sucrose (10%) | 265.1 | 0.664 |

**Table S11.** **Viscosity of blank and Cubosomal Gels at different HPMC concentrations (% w/w)**

| **Gel formulation code** | **% w/w HPMC** | **Viscosity (PaS)** |
| --- | --- | --- |
| Blank G1 | 1 | 3.89$\pm$0.69 |
| Blank G2 | 1.5 | 13.79$\pm$0.26 |
| Blank G3 | 2 | 49.03$\pm$1.38 |
| OF12_G1 | 1 | 6.45$\pm$0.49 |
| OF12_G2 (OF12-GEL) | 1.5 | 23.73$\pm$1.28 |
| OF12_G3 | 2 | 61.06$\pm$2.3 |
| OF16_G1 | 1 | 8.14$\pm$0.3 |
| OF16_G2 (OF16-GEL) | 1.5 | 23.63$\pm$1.46 |
| OF16_G3 | 2 | 71.09$\pm$1.7 |

**Table S12. The cumulative amount of 5-FU permeating at the end of 24 h (Q_24_), flux and drug content in the skin from free 5-FU solution and optimized formulations**

| **Systems** | **Flux (µg/cm^2^/h)** | **Drug permeated at the end of 24 h Q_24_ (µg)** | **Drug retained in skin (µg/cm^2^)** |
| --- | --- | --- | --- |
| 5-FU solution | 23.37 ± 0.18 | 543.54$\pm$5.15 | 114.14$\pm$3.41 |
| OF12 | 4.79$\pm$ 0.03 | 123.33$\pm$ 0.49 | 171.45$\pm$ 1.44^****^ |
| OF12-GEL | 8.98$\pm$ 0.03 | 220.15$\pm$ 0.5 | 323.71$\pm$ 4.25^****^ |
| OF16 | 3.41 $\pm$0.06 | 87.39$\pm$ 0.11 | 142.06$\pm$ 1.23^**^ |
| OF16-GEL | 4.34 $\pm$0.02 | 108.43$\pm$0.48 | 162.02$\pm$ 4.29^****^ |

Mean$\pm$SD, n=3; **p<0.001 ****p<0.0001 in comparison to pure 5-FU solution.

**Table S13.** **IC_50_ values pure drug, optimized formulations (OF12, OF16), and synthesized dimeric surfactants (GS12, GS16) in HaCat and A431 cell lines.**

| **Samples** | **IC50 (µg/mL)** | |
| --- | --- | --- |
|  | **HaCaT** | **A431** |
| Pure 5-FU | 1.44$\pm$0.07 | 1.15$\pm$0.02 |
| OF12 | 3.03$\pm$0.05 | 0.77$\pm$0.02 |
| OF16 | 2.82$\pm$0.16 | 1.04$\pm$0.11 |
| GS12 | 4.4$\pm$0.39 | 1.29$\pm$0.02 |
| GS16 | 4.05$\pm$0.13 | 1.68$\pm$0.03 |

**Table S14. Primary skin irritation studies of formulations in rats**

| **Skin response** | **Reaction grade observed** | | | | | | **Mean PII** |
| --- | --- | --- | --- | --- | --- | --- | --- |
|  | Rat 1 | | Rat 2 | | Rat 3 | |  |
|  | Erythema | Edema | Erythema | Edema | Erythema | Edema |  |
| Control | 0 | 0 | 0 | 0 | 0 | 0 | 0 |
| Blank | 0 | 1 | 0 | 0 | 0 | 0 | 0.16 |
| OF12-GEL | 1 | 0 | 0 | 1 | 0 | 0 | 0.33 |
| Positive control | 1 | 0 | 1 | 1 | 1 | 1 | 0.83 |

**Table S15. Histopathological grading after hematoxylin/ eosin staining in skin irritation study in rats**

| **Groups** | **Inflammatory cells** | **Edema** | **fibrosis** | **Hyperkeratosis** | **Ulcer/abscess** |
| --- | --- | --- | --- | --- | --- |
| **Untreated control** | **-** | **-** | **-** | **-** | **-** |
| **0.8% formalin** | **+++** | **-** | **-** | **-** | **+++** |
| **Blank formulation** | **-** | **-** | **-** | **-** | **-** |
| **OF12-GEL** | **-** | **-** | **-** | **-** | **-** |

**Table S16. Histopathological assessment of respective treatment groups**

| **Treatment Groups** | **Degree of Keratinisation** | **Nuclear Pleomorphism** | **Mitosis** | **Inflammatory Infiltration** |
| --- | --- | --- | --- | --- |
| **Group I** | 0 | 0 | 0 | 0 |
| **Group II** | 3 | 4 | 3 | 3 |
| **Group III** | 1 | 2 | 2 | 3 |
| **Group IV** | 0 | 0 | 0 | 2 |

Scale: 0 (Nil), 1 (mild), 2 (moderate), 3 (severe), 4 (very severe)

**Table S17. Number of survived animals and deaths recorded during the study**

|  | **Disease Control** | **Marketed formulation treated** | **OF12-GEL treated** |
| --- | --- | --- | --- |
| Survived animals | 0 | 0 | 3 |
| Deaths/Sacrificed | 5 | 5 | 2 |
| Median survival | 20 | 18 | Undefined |

**Table S18. Histological features and gradings of different groups**

| **Histological features** | **Sham control** | | **Disease control** | | **Marketed formulation** | | **OF12-GEL** | |
| --- | --- | --- | --- | --- | --- | --- | --- | --- |
|  | **21** | **22** | **4** | **5** | **9** | **10** | **14** | **15** |
| Stromal density | - | - | Severe | Severe | Marked | Marked | Moderate | Moderate |
| Tumor necrosis | - | - | Mild | Mild | Moderate | Moderate | Marked | Marked |
| Neo-angiogenesis | - | - | Severe | Severe | Moderate | Moderate | Moderate | Moderate |
| Inflammatory cells infiltration | - | - | Severe | Marked | Marked | Moderate | Moderate | Moderate |
| Desmoplasia | - | - | Severe | Marked | Marked | Moderate | Moderate | Moderate |
| Presence of mitotic figures | - | - | Marked | Severe | Moderate | Moderate | Moderate | Moderate |
| **Skin histology** | | | | | | | | |
| Dermal invasion | - | - | Marked | Severe | Marked | Marked | Moderate | Mild |
| Dermal inflammation | - | - | Marked | Marked | Moderate | Moderate | Moderate | Mild |
| Dermal congestion and neo-angiogenesis | - | - | Severe | Severe | Marked | Marked | Moderate | Moderate |

Lesions are graded as per: Minimal < Mild < Moderate < Marked < Severe

**Table S19. Microvessel density measurements**

| **Slide no.** | **Mean**$\boldsymbol{\pm}$**SEM MVD** |
| --- | --- |
| Disease control | 15.70$\pm$0.94 |
| Marketed formulation | 9.50$\pm$0.43 |
| OF12-GEL | 8.65$\pm$0.45 |

MVD/hpf: No. of blood vessels present per field under the high magnification (40x) view.

**Table S20. HPLC chromatographic conditions for the estimation of 5-FU**

| **Analyte** | 5-Fluorouracil (5-FU) |
| --- | --- |
| **Stationary phase** | Phenomenex LUNA C-18 (250.0 x 4.6 mm, 5 µm, 100Å) |
| **Mobile phase** | 98:2 water (pH 3.0) adjusted using TFA: ACN |
| **Detection wavelength** | 265 nm |
| **Flow rate** | 0.8 mL/min |
| **Injection volume** | 20 µL |
| **Column oven temperature** | 25 °C |
| **Autosampler temperature** | 5 °C |
| **Sample solution** | 5-FU in Phosphate Buffer pH 7.4 |
| **Run Time** | 1. min |

**Table S21. Results of validation parameters for 5-FU estimation for analytical method**

| **Validation parameters** | **Method responses** | **Acceptance criteria** | **Observed** |
| --- | --- | --- | --- |
| System suitability | RSD of peak area (n=6) | RSD < 2.0% | 0.18 |
|  | Tf_10%_ | < 1.5 | 1.43 |
|  | N | > 2000 | 4379.05 |
| Linear regression data | Linearity (µg/mL); (n=3) | **-** | 1 – 10 |
|  | Slope |  | 76150 |
|  | Y-intercept when X=0 |  | 16711 |
|  | R^2^ |  | 0.9987 |
|  | LOQ (ng/mL) | **-** | 100 |
|  | LOD (ng/mL) | **-** | 72 |
| Precision | %RSD for intraday and interday | < 2.0 % | 0.57 |
| Accuracy | Initial conc. (µg/mL) | Observed mean conc.(µg/mL); n=3 | Mean recovery (%) |
|  | 1 | 0.9 | 89.88 |
|  | 3 | 2.93 | 97.65 |
|  | 5 | 4.96 | 99.25 |

**Table S22. Results of validation parameters for bioanalytical method**

| **Validation parameters** | **Method responses** | **Acceptance criteria** | **Observed value** | |
| --- | --- | --- | --- | --- |
| System suitability | RSD of peak area (n=6) | RSD <2.0 % | 1.14 | |
|  | Tf_10%_ | <1.5 | 1.403 | |
|  | N | >2000 | 2979 | |
| Linear regression data | Linearity (ng mL^-1^); (n=3) | **-** | 150-10000ng | |
|  | Slope |  | 0.0014 | |
|  | Y-intercept when X=0 |  | 0.2624 | |
|  | R^2^ |  | 0.9971 | |
| Accuracy | Sample | **85-120%** |  | |
|  | LQC |  | 107.9 | |
|  | MQC |  | 102.9 | |
|  | HQC |  | 98.3 | |
| Precision | Sample | **%CV<15%** | Inter-batch | Intra-batch |
|  | LQC |  | 6.62 | 6.20 |
|  | MQC |  | 4.72 | 5.75 |
|  | HQC |  | 3.88 | 5.89 |

HQC- 10 µg/mL; MQC- 1 µg/mL; LQC- 250 ng/mL
